# Existence of Inverted Profile in Chemically Responsive Molecular Pathways in the Zebrafish Liver

**DOI:** 10.1371/journal.pone.0027819

**Published:** 2011-11-29

**Authors:** Choong Yong Ung, Siew Hong Lam, Xun Zhang, Hu Li, Jing Ma, Louxin Zhang, Baowen Li, Zhiyuan Gong

**Affiliations:** 1 Department of Biological Sciences, National University of Singapore, Queenstown, Singapore; 2 Department of Mathematics, National University of Singapore, Queenstown, Singapore; 3 Graduate School for Integrative Sciences and Engineering, National University of Singapore, Queenstown, Singapore; 4 Department of Physics and Centre for Computational Science and Engineering, National University of Singapore, Queenstown, Singapore; 5 Department of Biomedical Engineering, Boston University, Boston, Massachusetts, United States of America; Memorial Sloan Kettering Cancer Center, United States of America

## Abstract

How a living organism maintains its healthy equilibrium in response to endless exposure of potentially harmful chemicals is an important question in current biology. By transcriptomic analysis of zebrafish livers treated by various chemicals, we defined hubs as molecular pathways that are frequently perturbed by chemicals and have high degree of functional connectivity to other pathways. Our network analysis revealed that these hubs were organized into two groups showing inverted functionality with each other. Intriguingly, the inverted activity profiles in these two groups of hubs were observed to associate only with toxicopathological states but not with physiological changes. Furthermore, these inverted profiles were also present in rat, mouse, and human under certain toxicopathological conditions. Thus, toxicopathological-associated anti-correlated profiles in hubs not only indicate their potential use in diagnosis but also development of systems-based therapeutics to modulate gene expression by chemical approach in order to rewire the deregulated activities of hubs back to normal physiology.

## Introduction

Living organisms are constantly exposed to potentially harmful chemicals within their living habitats. Detoxification that involves metabolizing and eliminating these chemicals are of great importance to maintain healthy equilibrium. In vertebrates, the liver is an essential organ in detoxifying xenobiotics and monitoring nutritional cues via homeostatic regulation in broad biochemical processes. This offers the liver as an excellent organ to study homeostatic processes in response to chemical imbalances and xenobiotic insults.

Several omics studies had revealed that genes involved in xenobiotic metabolism, heat shock signaling, oxidative stress, cell proliferation, apoptotic pathway, nuclear receptor signaling and proteasome are among those mostly reported to show altered expression [1]–[5]. Some of these genes even show distinct differential expression profiles in response to specific chemicals [6]. These specific responsive genes are no doubt useful for biomarkers with predictive and diagnostic values [2], [4], [6]. However, large-scale omics-integrated studies correlating molecular pathways that are sensitively perturbed by various chemicals are still limited. This kind of study is important because deregulation of molecular pathways usually involved multiple genes that convey greater biological importance than just individual genes in the context of liver homeostasis.

Thus, it is important to understand the intricate relationship among molecular pathways in the liver under various chemical insults as their deregulated activities often resulting metabolic disorders, cancers, and liver damage. In this study we used the zebrafish model to identify molecular pathways that act as hubs to respond to perturbations triggered by different chemicals. We exposed zebrafish to six different chemicals, Benzo-[a]-pyrene (BAP), 17-β estradiol (E2), 4-Nitrophenol (NP), 4-Chloroaniline (CA), Arsenic (V) acid (As) and Mercury (II) chloride (Hg), at various concentrations and under different exposure durations, and generated hepatic transcriptomic data by DNA microarray. These chemicals were chosen because they serve as representatives of selected environmental toxicants and pollutants that are potential health hazards to various organisms including humans, hence having considerable public health concern. BAP is a polycyclic aromatic hydrocarbon that serves as a representative of persistent organic pollutants while E2 is a steroid that represents estrogenic compounds and these two classes of compounds are known endocrine disruptors found in the environment [7], [8]. Arsenic (V) represents a metalloid compound, and mercury (II) represents a heavy metal compound and both are ubiquitous environmental toxicants and their poisoning are public health issues worldwide [9], [10]. Both NP and CA are organo-nitrogen compounds of various industrial and agricultural sources that have polluted the environment [11], [12] and are known to cause hemoglobin adducts resulting in methemoglobinemia and possibly anemia [12], [13]. NP has also been reported to act as an endocrine disruptor [14] while BAP, E2 and arsenic (V) are also known to be genotoxic carcinogens [3], [4], [15], and mercury (II) can induce cellular damage particularly in central nervous system, kidney and liver [5], [16].

Using gene set enrichment, functional correlation and network analyses, we successfully defined a group of molecular pathways acting as hubs for their frequent respond to multiple chemicals and high degree of functional correlations (connectivity degrees) to many other molecular pathways. Interestingly, we found that these hubs were organized into two groups showing inverted activity correlation with each other. Our current work revealed that the inverted activity profiles of hubs are obvious only when a fish is confronted with chemically-induced toxicopathological conditions. Since the identified hubs play important roles in liver homeostasis, their malfunctions suggest broad association to liver disorders. Furthermore, analyses using independent external datasets obtained from liver tissues of rat, mouse, and human cells exposed to chemicals [17]–[19] also suggested the present of inverted activity profiles of hubs. This implies that the observed inverted activity profiles may be conserved from fish to mammals albeit further characterizations are required. The existence of these toxicopathological-associated anti-correlation in hubs open the possibility to devise combinatorial therapeutics with drug cocktails to rebalance these inverted profiles back to normal physiology.

## Results

### Molecular Pathways that are Frequently Perturbed by Chemicals in the Liver

We generated a total of 168 arrays (48 arrays from control groups, 120 arrays from the chemically treated groups) for zebrafish livers exposed to six different chemicals at various concentrations and durations, accounting for a total of 40 specific chemically-induced perturbations. For comparative study, we included 3 additional hepatic transcriptomic data obtained from zebrafish experiments with starvation and specific carbohydrate dietary manipulation [20], [21]. The overall 43 perturbations and treatment conditions are listed in the Table S1.

**Figure 1:**
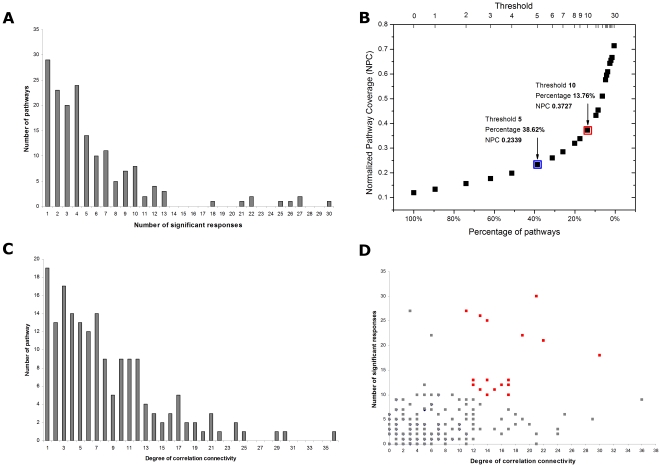
Responsiveness and connectivity of molecular pathways perturbed by chemicals. (**A**) Number of perturbed pathways to their respective number of significant responses across 43 distinct chemical perturbations. The graph was obtained by counting and grouping total number of 189 non-redundant molecular pathways that were significantly perturbed. Most pathways show low number of significant responds across multiple perturbations. Pathways showing same responsiveness and connectivity are plotted as one data point. (**B**) Normalized pathway coverage across 43 perturbations and percentage of pathways with a given cutoff threshold of significant respond. The thresholds used to define high and medium responsiveness are indicated in red and blue boxes, respectively. (**C**) Number of pathways with respect to their connectivity degree, as obtained from Pearson correlation analysis using NES scores across 43 perturbations as meta-activities of molecular pathways. Pairs of pathways with Pearson correlation coefficient (PCC)>0.5 or PCC<−0.5 are considered functionally connected, where the connectivity degree denotes number of functionally connected pathways to a given pathways. Most pathways show low number of functional correlations to other pathways across multiple perturbations. (**D**) Relation of the number of significant responds of pathways to their connectivity degree. Red spots are hubs with both high responsiveness (with at least 10 significant responds) and high degree of correlation connectivity (with at least 10 degree of connectivity). Non-hub pathways are indicated as gray spots. Most non-hub pathways show both low responsiveness and low degree of connectivity as most of them assembled at the lower left of the plot, with a smaller number of non-hubs with high degree of connectivity at lower right of the plot.

To examine whether the expression of genes within a molecular pathway is enriched by a perturbation, enrichment analyses considering the relative ranks of gene expression profile were conducted by Gene Set Enrichment Analysis (GSEA) [22], which utilizes Kolmogorov-Smirnov-like statistic. We performed GSEA by comparing each of the 40 chemically- and 3 dietary-induced perturbations against their respective control groups for pathways of at least 10 detected genes within each expression experiment. Pathways with nominal p-value<0.05 for a given enrichment were considered significant in response to a perturbation. We tabulated the z-score corrected GSEA results with their corresponding perturbations in a matrix in order to determine how frequent each molecular pathway was affected. The full GSEA results of 246 molecular pathways with minimal 10 genes for each perturbation experiment are presented in Data S1. Redundant pathways with poor responsiveness were subsequently omitted, leaving 189 non-redundant molecular pathways for further analyses. We plotted the number of pathways with their respective number of significant responses across 43 conditions (Figure 1A). Most of these pathways show low number of significant responses across multiple perturbations.

We next defined a function called normalized pathway coverage (NPC) in order to determine appropriate thresholds to partition pathways into categories composing of high responsive (HR), medium responsive (MR), and low responsive (LR) across multiple perturbations (see Materials and Methods). The relation of NPC against the percentage of pathways with the number of significant responses of i-th pathway≥threshold (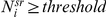) is plotted (Figure 1B). From the plot, we defined pathways with at least 10 significant responses (nominal p-value<0.05) across 43 perturbations as high responsive pathways (HR). Pathways with 5 to 9 and less than 5 significant responses are defined as medium responsive (MR) and low responsive (LR), respectively. There are approximately 61.4%, 24.9%, and 13.8% of LR, MR, HR pathways, respectively.

### Functional Correlations of Molecular Pathways Across Multiple Chemical Perturbations

At cellular level, activities among molecular pathways are governed by intricate regulation and expression of their genes. To investigate the correlation and potential functional connectivity of molecular pathways, we performed Pearson correlation to assess linear correlation among 189 molecular pathways across 43 perturbations. Normalized Enrichment Score (NES) generated by GSEA (see Materials and Methods) were used to represent the meta-activities of pathways. In general, the greater the NES value, the greater the enrichment for genes presented in a molecular pathway in a pre-ranked transcriptomic data of a perturbation. The negative or positive NES value denotes whether the pre-ranked transcriptomic data is positively (i.e. most enriched genes were up-regulated) or negatively (i.e. most enriched genes were down-regulated) correlated with a particular molecular pathway.

A pair of pathways is considered functionally connected if the calculated Pearson correlation coefficient (PCC)>0.5 or PCC<−0.5 (Data S2) with p-value<0.001 (Data S3). The number of non-redundant 189 pathways with their respective connectivity degree is plotted in Figure 1C, with average connectivity degree of 7.43. We defined pathways that exhibit degree of functional correlations (connectivity) larger than 10 (i.e., <30% of total 189 non-redundant pathways) as highly connected. There are 57 molecular pathways with ≥10 connectivity degrees, which were defined as highly connected pathways. Those pathways with less than 10 connectivity degrees were considered as low connected. Pathways associated with different category of responsiveness and connectivity degree are given in Table S2.

Results from Pearson correlation also revealed that pathways with known associated functions also show significant correlations in fish triggered by chemicals (Table S3). For examples, glycolysis and gluconeogenesis are expected to show high functional correlation as they share a substantial number of common genes. However, pathways such as glycolysis and pyruvate metabolism, glycolysis and citrate cycle as well as proteasome and ubiquitin-mediated proteolysis which share little or no common gene also show significant correlation for known associated functions. This indicates that NES generated from GSEA is a valid approximation to represent the overall pathway activities in a given biological state.

### Identification of Hubs with High Responsiveness and High Connectivity

We are interested in finding molecular pathways acting as hubs that they are not only frequently perturbed by various chemicals but also show high functional connectivity to many other pathways. We plotted the frequency of perturbations and the degree of connectivity in order to visualize their relationships for each of the molecular pathway (Figure 1D). Interestingly, most molecular pathways with low responsiveness tend to have poorer correlation (gray spots in Figure 1D) than those with high responsiveness. Hubs with both high responsiveness and high connectivity degrees are shown as red spots in Figure 1D. Molecular pathways with low responsiveness and poor connectivity are thought to be impervious to most chemical perturbations and are less likely to be co-regulated with other pathways during chemical insults. However, poor connectivity for low responsive pathways can also be due to the current limited dataset and lack of sensitivity of the assays used.

We identified a group of 16 hubs corresponding to 43 perturbations in this study (pink and green highlights in Table S2). Overall, these pathways are associated with lipid metabolism (fatty acid metabolism; mitochondrial fatty acid beta oxidation; PPAR signaling; propanoate metabolism; butanoate metabolism), amino acid metabolism (tryptophan metabolism; beta-alanine metabolisms; valine, leucine and isoleucine degradation), protein biosynthesis and degradation (ribosome; aminoacyl t-RNA biosynthesis; N-glycan biosynthesis; proteasome), neuroactive ligand receptor interaction, circadian exercise and IL6 pathway. Their ease of being perturbed and their high functional correlation with each other suggest that these hubs may play an important role in liver homeostasis towards broad chemical insults.

Molecular pathways related to hubs identified in this study had been found to be affected from both transcriptomics and proteomics works using other models and chemicals. For instance, amino acid metabolism, protein biosynthesis, fatty acid metabolism, fatty acid β-oxidation are affected in hepatic transcriptome and proteome of zebrafish treated with 17α-ethynylestradiol [23], [24]. Hepatic transcriptome and proteome from mice treated with propiconazole revealed differential expression and amount of proteins involved in amino acid metabolism including tryptophan metabolism, valine, leucine, and isoleucine degradation, together with lipid metabolism as well as proteasome [25]. Also, several transcriptomics and proteomics studies showed that signaling, mitochondrial function, lipid metabolism, amino acid metabolism, and energetic metabolism are affected in acetaminophen-treated mice and rats [26]–[28]. Besides, most serum N-linked glycoproteins are synthesized via N-glycan biosynthesis and secreted by the liver. Malfunction of N-glycan biosynthesis hence altered the N-linked glycoproteome that is known to be associated with chronic diseases such as hepatocellular carcinoma [29], [30]. In addition, the circadian pathway regulates several rate-limiting factors in vital cellular processes including lipid and sugar metabolism [31]. Furthermore, it had been known that alteration of excitatory and inhibitory amino acids relevant to neuroactive roles to hub neuroactive ligand receptor interaction affect hepatic encephalopathy during acute liver failure [32]. PPAR signaling, another hub identified in this study, is also shown to regulate liver repairing process where its malfunction impair liver regeneration [33]. Thus, hubs identified in this study appear to modulate liver homeostasis across broad cellular perturbations in higher vertebrates as well.

### Network of Hubs Shows Two Groups of Molecular Pathways with Inverted Functional Correlations

**Figure 2:**
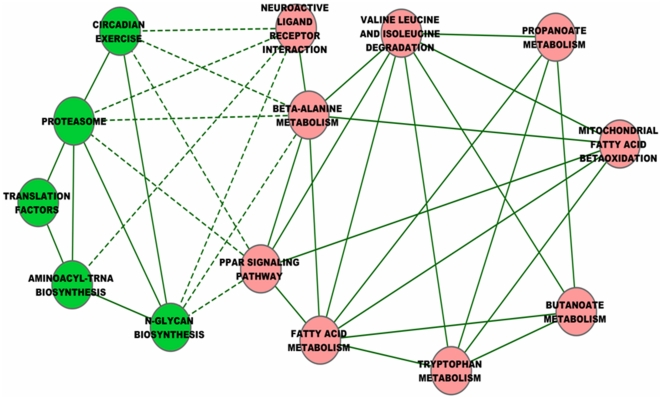
Network of hubs (Pathways with both high responsiveness and high connectivity). The network was constructed from Pearson correlation analysis using NES scores across 43 perturbations as meta-activities of molecular pathways. Pathways with Pearson correlation coefficient (PCC)>0.5 or PCC<−0.5 are considered connected to each others. Pink nodes (Group A) of hubs compose mostly lipid-related pathways and green nodes (Group B) of hubs are non-lipid pathways. Solid and dash lines indicate positive and negative correlations, respectively.

Using NES scores as meta-activities of molecular pathways, we performed Pearson correlation studies to capture their functional associability across 43 chemical perturbations. Hubs that are functionally correlated to one another are connected in network as shown in Figure 2. We noted that lipid metabolism together with amino acid metabolism and ribosome pathways were interconnected with positive correlations within one group (defined as Group A, connected with solid lines among pink nodes in Figure 2). Hubs from Group A show negative correlations to hubs in the other group (defined as Group B, connected with dash lines to green nodes in Figure 2). Hubs within the same group only show positive correlations whereas none of member within Group A or B shows positive correlation to hubs at the opposite group. Negative correlations are only observed to members between opposite groups. These observations indicate a clear inverted relationship between members of these two groups during chemical insults: pathways with positive correlations appear to be co-activated or co-repressed under a given biological state whereas pathways that show negative correlations appear to function oppositely under the same biological state. Hubs such as Ribosome and IL6 pathways are not included as they do not show inverted functionality to opposite group of hubs (discussed in the next section).

### Inverted Activity Profiles of Molecular Pathways are only Associated to Toxicopathological States

**Figure 3:**
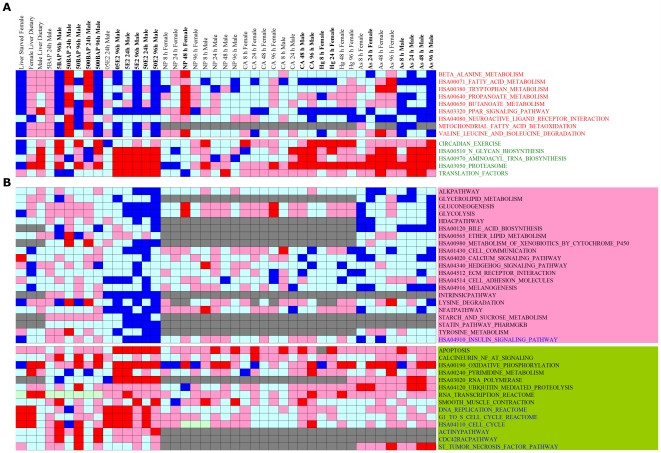
Meta-activity profiles of anti-correlated molecular pathways across 43 chemical perturbations. (**A**) Red and green fonts indicate hubs of Group A and B, respectively. Red and blue shades denote activation and suppression of pathways, as indicated from their respective NES scores, respectively. Darker shades correspond to their respective colors denote significant responds. Bolded sample labels are those conditions showing inverted activity profiles for hubs in Group A and B. (**B**) Meta-activity profiles of non-hub pathways showing anti-correlated behavior. Pathways in pink and green boxes are non-hub pathways with high anti-correlation scores associated to Group A and B, respectively (See Materials and Methods for detail description). Blue fonts are “outliers” pathways as discussed in the text. As = Arsenic (V); BAP = Benzo-[a]-pyrene; CA = Chloroaniline; E2 = Estradiol; Hg = Mercury (II) chloride; NP = 4-Nitrophenol. 8 h, 24 h, 48 h, and 96 h denote chemical exposure period for 8, 24, 48, and 96 hours, respectively.

The overall meta-activity profiles of hubs as represented by NES scores across 43 chemical perturbations is given in Figure 3A. Red and blue shades represent activation and suppression of pathway, respectively, with those significant responses are shaded with their respective darker colors. Gray shades indicate no enrichments found for a given condition.

As shown in Figure 3A, when fish were exposed to higher concentrations of chemicals (50BAP 24 h Male, 500BAP 24 h Male) or prolonged exposure periods (500BAP 96 h Male, 50E2 96 h Male, CA 96 h Male, As 96 h Male in Figure 3A), the inverted activity profiles between hubs became obvious for most of these perturbations (bolded sample labels in Figure 3A). We observed that when fish were perturbed under a physiological change (Liver Starved Female, Female Liver Dietary, Male Liver Dietary) or by short exposure periods to lower concentrations of chemicals (5BAP 24 h and 05E2 24 h Male), there was no obvious inverted activity profile among hubs.

We next devised a simple scoring scheme in order to assess whether there are other non-hub pathways that also exhibit anti-correlated behavior using 20 conditions with obvious inverted activity profiles (bolded sample labels in Figure 3) as “seeds” (see Materials and Methods for detail description). The identified anti-correlated pathways and their assigned “anti-correlation scores” are given in Table S4. As shown in Table S4, hubs ribosome and IL6 pathways are not belonged to anti-correlated pathways due to their low anti-correlation scores. The network for these anti-correlated pathways is given in Figure S1. There are 7 “outliers” that do not show connectivity (i.e., PCC<0.5 or PCC>−0.5) to their assigned group. For instance, insulin signaling pathway of Group A does not connect to any pathway in Group A but is negatively connected to Group B. The anti-correlation of these “outliers” may be mediated via weaker functional associations to pathways of the same positive/negative group. Similar to hubs, none of these anti-correlated non-hub pathways, including “outliers”, show negative correlation to members within the same group.

The overall meta-activity profiles of non-hub pathways showing anti-correlation are given in Figure 3B. There is no obvious inverted profile between non-hub pathways at both Group A and B at physiological, short exposure periods and lower concentrations of chemicals, except at one physiological condition (Female Liver Dietary). At higher chemical levels and prolonged exposure periods, the inverted profiles of non-hub pathways became more obvious albeit less prominent than hubs.

As shown in Figure 3, both male and female fishes show poor response to 4-nitrophenol (NP) treatments. This is also the case for chloroaniline (CA) treatments in female but not in male fish. It remains to be determined whether these chemicals can trigger inverted profile at higher concentrations. However, both sexes of fish exhibit inverted profiles of pathway under toxicopathological states although female fish show better tolerance to chemical treatments as suggested from their weaker inverted profiles (e.g. Arsenic profiles).

**Figure 4:**
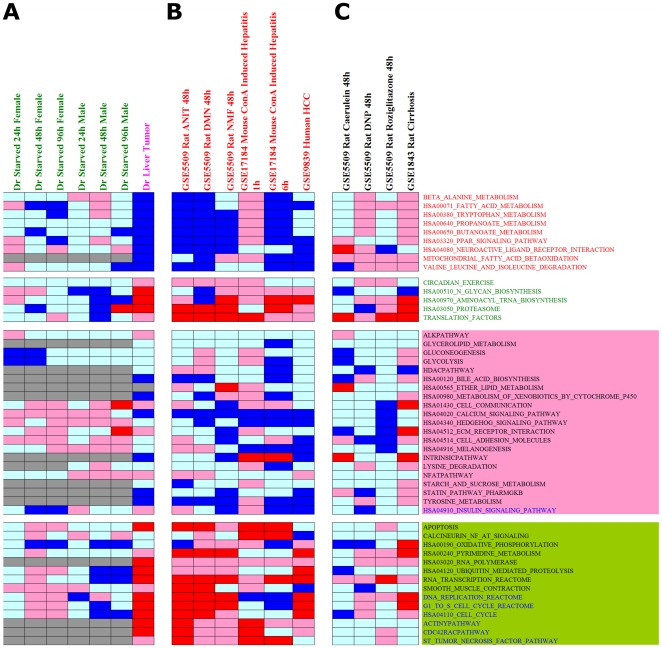
Meta-activity profiles of independent evaluation to investigate the association of inverted activity profiles with toxicopathological states. (**A**) Control data in this work where fishes were starved from 8 h to 96 h were used with data of 8 h starvation as comparative control to respective sexes of fish (green sample labels). No inverted activity profile is observed for starvation in hubs. However, carcinogen-induced liver tumor in zebrafish (pink sample label) generated from our previous work (GEO: GSE3519) [34] exhibits obvious inverted activity profile. (**B**) Expression data showing inverted profiles obtained from chemical-treated or pathologic states in liver tissues of rat, mouse, and human. (**C**) Expression data not showing inverted profile in chemical-treated and pathologic state in rat. Dr, *Danio rerio* (zebrafish); ANIT, Alpha-naphthyl-isothiocyanate; DMN, Dimethylnitrosamine; NMF, N-methylformamide; ConA, Concanavalin A; DNP, Dinitrophenol; HCC, Hepatocellular carcinoma.

To evaluate whether the inverted activity profiles are only associated to toxicopathological conditions induced by chemicals, we performed analyses using control data from our experimental works where fish were starved during the 96-hour of chemical treatments and samples were collected at 8 h, 24 h, 48 h and 96 h. Untreated male and female fish collected at 8 h were used as comparative controls for those starved for 24 h, 48 h, and 96 h for their respective sexes. As shown in Figure 4A, none of the starved fish show inverted activity profiles in hubs (green sample labels, Figure 4A) although male fish starved at 96 h show weak inverted activity profiles in non-hub pathways. We also analyzed transcriptomic data from carcinogen-induced liver tumor in zebrafish generated from our previous study [34]. We found that carcinogen-induced liver tumor in the zebrafish indeed showed obvious inverted activity profile in both hubs and non-hub pathways. Our analyses thus indicate, at least for data in the current study, that inverted activity profiles of molecular pathways especially hubs are associated to toxicopathological states.

### Inverted Activity Profiles of Molecular Pathways are also Present in Mammalian Systems

To explore the existence of these inverted activity profiles in other biological systems, we performed GSEA analyses for independent external hepatic transcriptomic data obtained from the Gene Expression Omnibus (GEO) for rat, mouse, and human treated with various chemicals. Four expression series, GEO:GSE5509 [17], GEO:GSE17184 [18], GEO:GSE9839, and GEO:GSE1843 [19] were used. Results showing the presence and absence of inverted activity profiles are in Figure 4B and Figure 4C, respectively. Expression data from three toxicants, alpha-naphthyl-isothiocyanate (ANIT), dimethylnitrosamine (DMN), and N-methylformamide (NMF), that are known to cause obvious histopathologic effects in rat liver [18], showed the existence of inverted activity profiles of molecular pathways as observed in the zebrafish (Figure 4B). Interestingly, additional expression data of three “non-toxic” compounds, caerulein, dinitrophenol (DNP), and rosiglitazone [17], did not exhibit inverted activity profiles (Figure 4C).

Analysis of transcriptomic data GEO:GSE17184 [19] from concanavalin A (ConA)-induced mouse fulminant hepatitis also suggest the presence of inverted activity profiles among molecular pathways observed in the zebrafish (Figure 4B). As ConA is commonly used to induce immune-mediated liver diseases in experimental models, the existence of these inverted profiles implicates their association to toxicopathological states. In addition, mouse treated with ConA for 6 h showed more obvious inverted profiles, especially in hub pathways, than those treated for 1 h, indicating that these inverted activity profiles are also associated to the progression of toxicopathological stages (Figure 4B). Moreover, transcriptomic data (GEO:GSE9839) from human liver tissue showing hepatocellular carcinoma (HCC) as a result of overexpression of HOX A13 exhibited inverted activity profiles, albeit more obvious among non-hub pathways (Figure 4B).

### Preferential of toxicopathological states in association towards Scheme B profile

We found most toxicopathological-associated states studied thus far are biased towards profile of “Scheme B”, with most hubs in Group B are activated accompanied with suppression of hubs in Group A (Figure 3 and Figure 4A). This is also the case for the mammalian systems (Figure 4B). We observed two conditions where male fish was exposed to 50 µg/L and 500 µg/L of BAP for 24 h (50BAP 24 h Male and 500BAP 24 h Male in Figure 3) showed inverted activity profiles of “Scheme A”, with members within Group A being activated. Female fish exposed to NP for 48 h (NP 48 h Female in Figure 3) also showed weak signal of Scheme A. Whether there are more toxicopathological states associated to Scheme A remained to be seen. At this stage, we are unable to determine the molecular mechanism for the preference of most toxicopathological states, at least from data in our current study, towards inverted activity profiles for Scheme B. Further works are needed to explore their regulatory mechanisms that lead to this preference.

## Discussion

In this study, we used zebrafish as a vertebrate model to identify hub molecular pathways that respond to multiple chemical perturbations in the liver and assess their functional correlations. We observed a substantial number of hubs involved in lipid metabolism. This is consistent with the fact that the liver plays a central role in lipid metabolism in response to nutritional cues by regulating the synthesis, oxidization, transport and excretion of lipids [35]. Also, signals from autonomic nervous system such as neuroactive ligand receptor interaction pathway can also alter metabolic states in the liver [36]. It had been noted that lipid accumulation is the common consequence associated to metabolic syndromes such as type 2 diabetes mellitus, hyperglycemia, hyperinsulinemia, cardiovascular diseases, and obesity [37]. This suggests that lost of homeostatic resiliency in lipid metabolism can lead to broad range of hepatic disorders such as steatosis, non-alcoholic steatohepatitis, cirrhosis, hypertension, and hepatocellular carcinoma [38].

Other hubs such as ribosome, proteasome, circadian exercise, N-glycan biosynthesis, aminoacyl-tRNA biosynthesis, translation factors, and IL6 pathway form non-lipid metabolic category. Suppression of proteolysis is normally required when a rapid transcriptional response and protein synthesis are needed in a cell such as during cell division. Translation factors, aminoacyl-tRNA and ribosomes are anticipated to coordinate at the subsequent stage after the transcription step. Also, circadian clock had been reported to couple with proteasome [39] to play essential roles in mediating nutritional metabolism in liver [40]. Besides, glycoproteins at the cell surface are found to modulate homeostatic responses. Malfunctions in N-glycan biosynthesis can thus leads to disorders such as autoimmunity, metabolic syndrome, aging, and even cancers [29], [41].

It had been reported that amino acids such as isoleucine, leucine, tyrosine, phenylalanine, tryptophan, lysine, and arginine are capable of inhibiting the chymotrypsin-like activity of the proteasome in a dose-dependent manner [42]. Our results show negative correlation of proteasome to beta-alanine metabolism which in turn positively correlated to valine, leucine and isoleucine degradation and tryptophan metabolism (Figure 2), implicating homeostatic roles of amino acids in protein degradation via proteasome machinery. In general, hubs identified in this study appear to play important homeostatic roles in the liver and their malfunctions can cause wide range of disorders.

**Figure 5:**
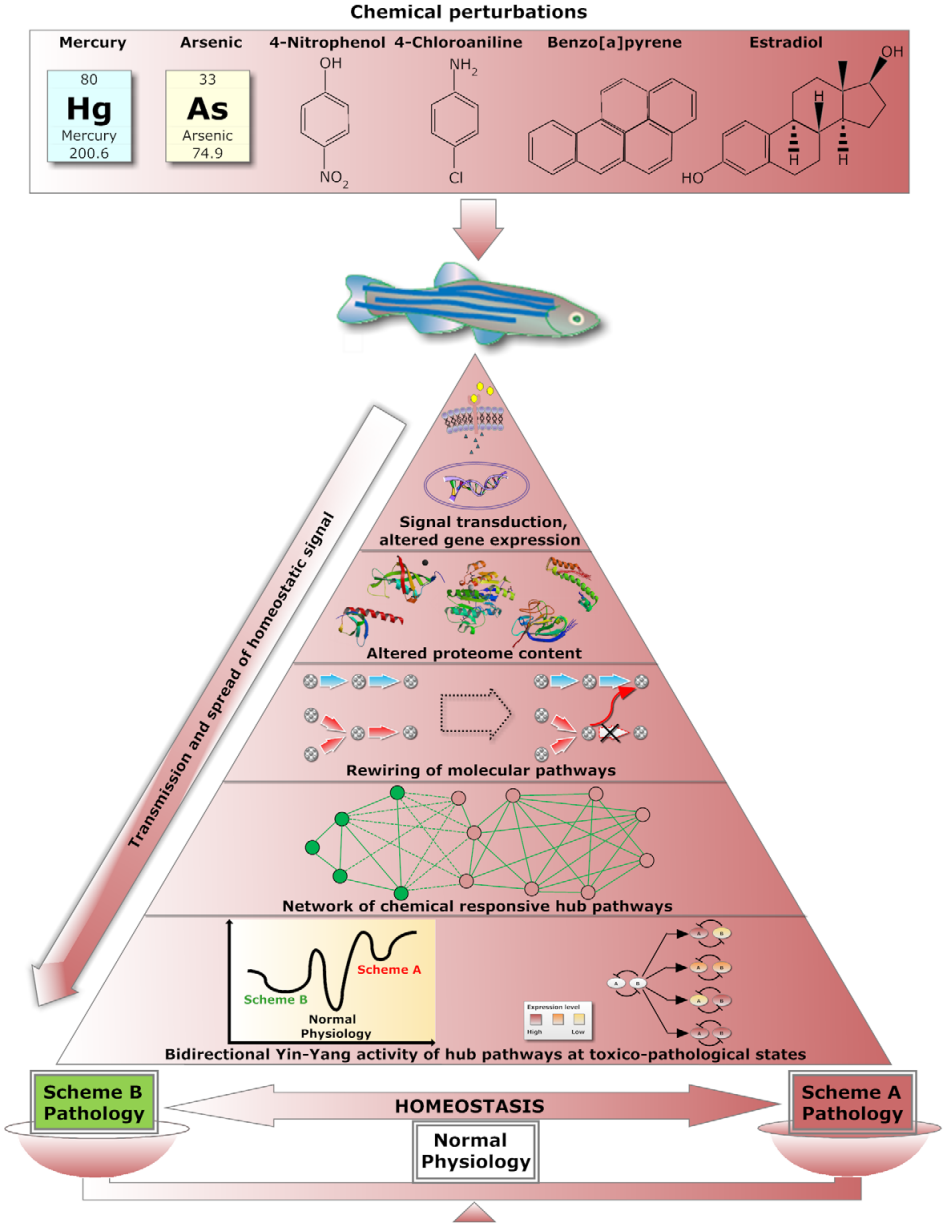
Schematic representation of overall finding and hypothesis in this work. Zebrafish was exposed to six different chemicals at varying concentrations and exposure durations. Upon exposure to these chemicals, the underneath homeostatic process as schematically illustrate in the pyramid will be initiated. The arrow beside illustrates the spread of homeostatic signals from smaller molecular scale to larger scale composing pathway networking. These chemicals will trigger conformational changes of signaling receptors and enzymes, resulting differential gene expression causing altered proteome content. If the chemicals are above certain threshold levels that a fish can cope for maintaining its normal physiological equilibrium, rewiring of molecular pathways via deregulated gene expression can take place leading to inverted profiles of hubs. The inverted functionality of hubs can be perceived as a two-component bistable switch that toggles a cell from one state to another, leading to toxicopathological states at either Scheme A (activation of hubs among Group A with suppression of hubs among Group B) or Scheme B (activation of hubs among Group B with suppression of hubs among Group A), depending on how the overall molecular rewiring drives the cell in navigating the toxicopathological landscape.

Figure 5 summarizes the overall outline and working hypothesis based on this work. The pyramid shows different levels of mechanisms of homeostatic processes upon perturbations induced by various chemicals. The arrow beside the pyramid indicates the flow and spread of homeostatic signal from the tip at the molecular level to the base consisting of molecular pathways that formed the homeostasis system in the liver. Upon receiving a chemical cue, a cascade of signal transduction coupled with chemical-induced conformational change of receptors and enzymes will be triggered. Expression of genes can be altered if the chemical signal is more intense than the threshold. This will lead to the next tier of regulation with altered levels of proteins including signaling molecules and transcription factors to subsequently drive rewiring of gene expression within hubs [43], [44]. The overall mechanisms of homeostasis can thus be metaphorically perceived as a dynamic balance that continuously adjusting biological processes in a liver cell to maintain healthy equilibrium within its tolerable limits.

The concept of “attractor” that is used to describe the behavior of complex systems in physics [45] had been adopted in stem cell biology to elaborate the pluripotency of a stem cell in navigating through a complex attractor landscape which ultimately leads to cell fate decision [46]. “Attractor” serves as a driving force to drive a stem cell towards a particular differentiated state upon receiving a differentiation factor. The same principle can be adopted in chemically-induced toxicopathological states. When a cell encounters a harmful chemical, homeostasis involving xenobiotic metabolism and its transport will be initiated. If the toxic level is above the threshold that a cell can cope within its healthy equilibrium, “attractor” resulting from deregulated gene expression will drive the affected cell to a particular toxicopathological state, depending on cellular context and toxic level exerted by a given chemical.

To maintain healthy equilibrium, a cell undergoes homeostasis processes constituting multiple tiers of negative and positive feedback loops to ensure all processes are tightly regulated such that all cellular processes only take place in the right amount, right place, and right time within a cell. The balanced activities of these negative and positive feedback loops can thus be viewed as balanced of “Yin” and “Yang” components, an ancient Chinese concept refers to opposite but mutually interdependent elements. In biology, this Yin-Yang concept had been utilized to describe the balance of various cellular activities, such as antioxidation/oxidation [47] as well as the balanced actions between oncogenes and tumor suppressor genes [48]–[50]. In this study, we found that inverted activity profiles of hubs were triggered only under conditions where a fish and mammalian cells were exposed to higher concentrations of chemicals, prolonged exposure durations, and carcinogen-induced tumors. As these conditions are associated to toxicopathological states, we therefore concluded that the inverted activity profiles of hubs, as can be metaphorically perceived as imbalanced Yin-Yang states, are of toxicopathological origin.

There are two toxicopathological schemes associated to the inverted profiles. “Scheme A” with activated Group A hubs and suppressed Group B hubs. The vice versa is applicable for “Scheme B”. Our current analyses indicate that most toxicopathological states favor Scheme B for fish and mammalian systems. We envisage lower “pathological barrier” (with analogy to energy barrier of enzymatic catalysis) for Scheme B across an imaginary toxicopathological landscape with normal physiological equilibrium being a global minimum (Figure 5). Also, Scheme B pathology exhibits “wider well” to represent more local states to trap a malfunctioning cell. On the other hand, Scheme A pathology, with much higher “pathological barrier”, is less likely to attain. Although this imaginary toxicopathological landscape provides qualitative explanation for inverted profiles of hubs in favor of Scheme B, further works are needed to unveil their exact molecular mechanisms.

The double-headed arrow at the base of the pyramid in Figure 5 indicates transitional reversibility and resiliency from normal physiology to both schemes of toxicopathological states (Scheme A or Scheme B) or vice versa. As hubs are associated with two groups within a network showing inverted functional correlations, this topology can be perceived as mutually inhibitive two-component system that can act as a toggle-like bistable switches to flip a cell from one state to another. Recent advances in network medicine [51] that aim to identify genes located within a disease module together with network pharmacology [52], [53] are now shaping drug development in the context of network and systems biology. Formulation using drug cocktails such as “long-tail drugs” [54] that aim to target a wide spectrum of gene activities can be the future trend in therapeutic design to modulate diverse biological processes.

Although we observed the association of toxicopathological states to inverted activity profiles of shared molecular pathways identified from fish to mammals, at this stage, we cannot exclude the possibility that there are other hub pathways showing inverted activities but fail to capture in this study. Furthermore, it is possible that there are totally different sets of molecular pathways showing inverted activities owing to different toxicopathological mechanisms under particular conditions. For instance, as shown in Figure 4C, we fail to capture inverted profiles from transcriptomic data of cirrhotic rat (GEO:GSE1843) [19] probably due to different pathological mechanism from our current analyses. Also, gene expression is context-dependent and the responsiveness of genes is affected by a number of factors such as chemicals, dosage, exposure period, tissues, species of animal model, as well as sex and age. Thus, it is necessary to extend current study by incorporating transcriptomic data with broader coverage of compounds in future in order to recover molecular pathways showing robust inverted activities under wide chemical and pathological perturbations. Notwithstanding at the preliminary stage, our finding for the existence of inverted activity profiles of molecular pathways that are associated to toxicopathological states in the zebrafish liver and mammalian systems may extend the direction of future researches in understanding molecular regulations in pathology as well as system-based therapeutics to treat complex diseases such as metabolic syndromes, cardiovascular diseases and cancers using appropriate experimental models.

## Materials and Methods

### Chemical treatment and sampling of zebrafish

Adult zebrafish (6 months–1 year old) were obtained from a local fish farm. The fishes were allowed to acclimatize in aquaria for several days before transferred into smaller containers for chemical exposure. Fishes were exposed to chemicals for up to 96 h at a density of one fish/200 ml at 27°C in a static condition. The treatment regimens used are summarized in Table S1 and were based on our previous experience with these chemicals which were determined based on published data available on Toxnet (http://toxnet.nlm.nih.gov/) and our preliminary acute toxicity exposure experiments conducted for the compounds [3]–[5]. De-chlorinated water and chemicals were renewed daily. All treatments were conducted using triplicate groups of four fishes. Liver samples were snap-frozen in liquid nitrogen and stored at −80°C prior to RNA extraction. All experimental protocols were approved by Institutional Animal Care and Use Committee (IACUC) of National University of Singapore (Protocol 079/07).

### Total RNA extraction and microarray hybridization

Total RNA was extracted using Trizol reagent (Invitrogen, USA) according to the manufacturer's instructions. Reference RNA for microarray hybridization was obtained by pooling zebrafish whole adult male total RNA with female total RNA at 9∶1 ratio. The integrity of RNA samples was verified by gel electrophoresis, and their concentrations were determined by UV spectrophotometer.

Reference RNA was co-hybridized with RNA samples either from chemically treated or control fish on a glass array spotted with 16.5K and 23K zebrafish oligo probes. Both reference and sample RNAs were reverse-transcribed and labeled differently using fluorescent dyes Cy-3 or Cy-5. After hybridization at 42°C for 16 hours in hybridization chambers, the microarray slides were washed in a series of washing solutions (2× SSC with 0.1% SDS; 1× SSC with 0.1% SDS; 0.2× SSC and 0.05× SSC; 30 seconds each), dried by low-speed centrifugation and scanned for fluorescence detection using the GenePix 4000B microarray scanner (Axon Instruments). More detailed protocols for microarray experiment and data acquisition have been described recently by us [55], [56].

### Description of hepatic transcriptomic data of zebrafish

All data used in this study is given in Table S1. Two series, GEO:GSE11107 [20] and GEO:GSE8856 [21] experimented with starvation and carbohydrate dietary manupulations were collected from Gene Expression Omnibus (GEO). The rest of data were generated from our in house experiments as summarized in Table S1.

### Microarray data processing and gene set enrichment analysis

The raw microarray data was normalized using Lowess method in the R package (http://www.braju.com/R/). The zebrafish genes were mapped to human homologous genes as previously described [34]. Student's t-test was performed to evaluate statistical significance between treatments and controls. The “GSEAPreranked” option of GSEA [22] was used for gene set enrichment analysis. The ranking metric used was log_10_(1/P) where P is the p-value of a gene obtained from Student's t-tests. Positive and negative values of log_10_(1/P) were assigned for up- and down-regulated genes, respectively. The genes were then ranked in descending order based on values of log_10_(1/P). The ranked genes were the input array for GSEA and were mapped to 639 pre-defined gene sets of canonical pathways obtained from the GSEA website (http://www.broadinstitute.org/gsea/). Gene sets with less than 10 genes mapped to input were excluded from further analysis.

GSEA uses Kolmogorov-Smirnov statistic to evaluate the statistic significance of genes to which a pre-defined gene set is overrepresented at the top or bottom rank of whole transcriptome profile [22]. An enrichment score (ES) for each pathway was calculated by walking down the ranked profile, increasing a running-sum statistic when a gene in a pre-defined gene set was encountered and decreasing it when the gene was absent. The ES is the maximum deviation from zero encountered in the random walk corresponds to a weighted Kolmogorov-Smirnov-like statistic. The statistical significance of a given ES was estimated by using an empirical phenotype-based permutation test procedure (1000 permutations were used). The phenotype labels were permutated and the ES of the gene set for the permutated data were recomputed to generate a null distribution for the ES. The empirical, nominal p-value of the observed ES was then calculated in relative to this null distribution. The estimated significance level was adjusted with multiple hypothesis testing. The ES for each gene set was first normalized to the size of the set yielding a normalized enrichment score (NES) with the following relation:
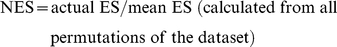
Pathway with nominal p-value<0.05 was considered significantly respond to a perturbation. Positive and negative values of NES indicate that pathways were activated and suppressed, respectively.

### Definition of pathway responsiveness

In order to determine appropriate thresholds (number of significant responses) for partitioning pathway category corresponding to high responsive (HR), medium responsive (MR), and low responsive (LR), we defined a function called normalized pathway coverage (NPC) for one particular chosen threshold as
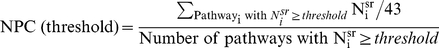
where 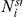 is the number of significant responses (p-value<0.05) of 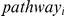 under 43 conditions (for details, see the demonstration in Figure S2). Normalized pathway coverage represents the average contribution of significant responses from each pathway with 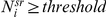. For example if NPC = 0.3, it means for average there are 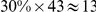 significant responses can be observed for one specific pathway. Higher value of normalized coverage for high responsive (HR) pathway is expected. Also, the percentage of pathways with 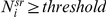 is another factor to determine appropriate thresholds. Therefore, we try to balance the NPC and number of pathways in the process of defining pathway responsiveness. Correlation between normalized pathway coverage (NPC) and percentage of pathways with 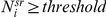 is plotted in Figure 1B.

### Use of correlation method to assess functional association of pathways

In this study, we used NES obtained from GSEA to represent the “meta-activity” of a pathway. We used Pearson correlation which assumes linear dependence between variables to infer functional association between pathways. Matrix of z-score-corrected NES values was used as input to calculate Pearson correlation coefficient (PCC) as stated below:
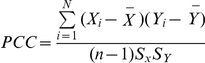
where 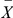 and 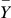 are means of X and Y respectively, 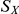 and 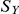 are standard deviations of X and Y respectively, and n is the number of pathways.

To evaluate the significance between correlations, a matrix of p-values for testing the hypothesis of no correlation was performed. Each p-value is the probability of getting a correlation as large as the observed value by random chance, where the true correlation is zero. We considered the correlation between pathways is significant if PCC>0.5 or PCC<−0.5 with p-value<0.0001.

### Devising a simple scoring scheme to identify anti-correlated pathways

We observed there are 20 conditions in our data showing obvious inverted activity profiles among hubs (bolded labels in Figure 3A). We thus used these 20 conditions as “seeds” to identify other pathways that may also exhibit inverted profiles. For these 20 conditions, if a significant respond of a given pathway is encountered, +1 score is assigned to the group (A or B, with at least three hub pathways) that shows similar profile with a particular pathway, else a penalty of −1 score is assigned. Accumulative scores for Group A (A_score) and B (B_score) for this pathway are calculated. A pathway will be assigned as member of Group A if its A-score≥+3 (B-score≤−3) and vice versa. To assess whether these assigned pathways are really anti-correlated, “anti-correlation score” for a pathway is calculated as followed:
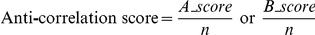
whichever A_ or B_score is positive, and n is the number of significant responds of a pathway across 20 anti-correlated conditions and 3≤n≤20. A pathway is considered anti-correlated if the anti-correlation score >0.7.

### Data processing of independent mammalian datasets

In order to assess the present of inverted activity profiles of molecular pathways in mammalian systems, we collected independent external hepatic transcriptome data of rat and mouse treated with chemicals from the Gene Expression Omnibus (GEO). Transcriptome data of human hepatocellular carcinomas was also included. Four GEO expression series, GEO:GSE5509 [17], GEO:GSE17184 [18], GEO:GSE9839, and GEO:GSE1843 [19] were used. Student's t-test was performed for each series between treated with their respective control groups. log_10_(1/P) was computed for each gene and was used as ranking metric as described in the zebrafish data. Gene symbols provided by platforms in these selected GEO series were used as input for GSEA, with molecular pathways less than 10 genes mapped to the ranked input arrays were discarded. Pathways with nominal p-value<0.05 were considered significantly affected. Molecular pathways showing inverted profiles in zebrafish were selected to assess their activity profile in mammalian systems.

## Supporting Information

Figure S1 **Network of molecular pathways showing anti-correlated behavior.** Hubs are indicated with red and green fonts. Pink and green nodes represent pathways associated to Group A and B, respectively.
(TIF)

Figure S2
**Demonstration of Normalized Pathway Coverage (NPC) function, which represents the average contribution of significant responses from each pathway with** 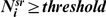 **.** Lets us assume that the number of significant responses of three pathways P_i_, P_j_, P_k_, are larger or equal to a threshold, say 5, then the NPC for these pathways of threshold 5 will be NPC(5) = (5/43+7/43+6/43)/3≈0.1395. Similar computation is performed for other pathways with different values of thresholds. The computation results are shown in the figure for 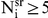. Crosses (x) are pathways with 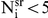 where they are not used for computation. Ticks (√) are pathways that fulfilled the threshold criteria for further computation.(JPG)

Data S1 **z-score corrected normalized enrichment scores for each molecular pathway across 43 chemical perturbations.**
(XLS)

Data S2 **Pairwise Pearson correlation between pathways.**
(XLS)

Data S3 **Statistical test (p-value) for pairwise correlation between pathways.**
(XLS)

Table S1 **Categories, types, and experimental summary of hepatic transcriptomic data used in this study. As = Arsenic (V); BAP = Benzo-[a]-pyrene; CA = Chloroaniline; E2 = Estradiol; Hg = Mercury (II) chloride; NP = 4-Nitrophenol.**
(DOC)

Table S2 **Categories of pathways at different levels of responsiveness and degree of connectivities. HR = Highly responsive; MR = Medium responsive; LR = Low responsive; HC = Highly connected; LC = Low connected.**
(DOC)

Table S3 **Categories of pathways with known associated functions showing significant correlation coefficients.**
(DOC)

Table S4 **Assigned anti-correlation scores to pathways showing significant responds to 20 selected conditions with obvious inverted activities of hubs as “seed”.** Red and green fonts are hub pathways of Group A and B, respectively. Pink and green backgrounds are anti-correlated pathways assigned to Group A and B, respectively. See Materials and Methods for detail description for anti-correlation scores.
(DOC)
