# Supplementary figures and images for: Existence of Inverted Profile in Chemically Responsive Molecular Pathways in the Zebrafish Liver
Source: PLoS One. 2011 Nov 29;6(11):e27819. doi: 10.1371/journal.pone.0027819 (PMC3226580; doi:10.1371/journal.pone.0027819)

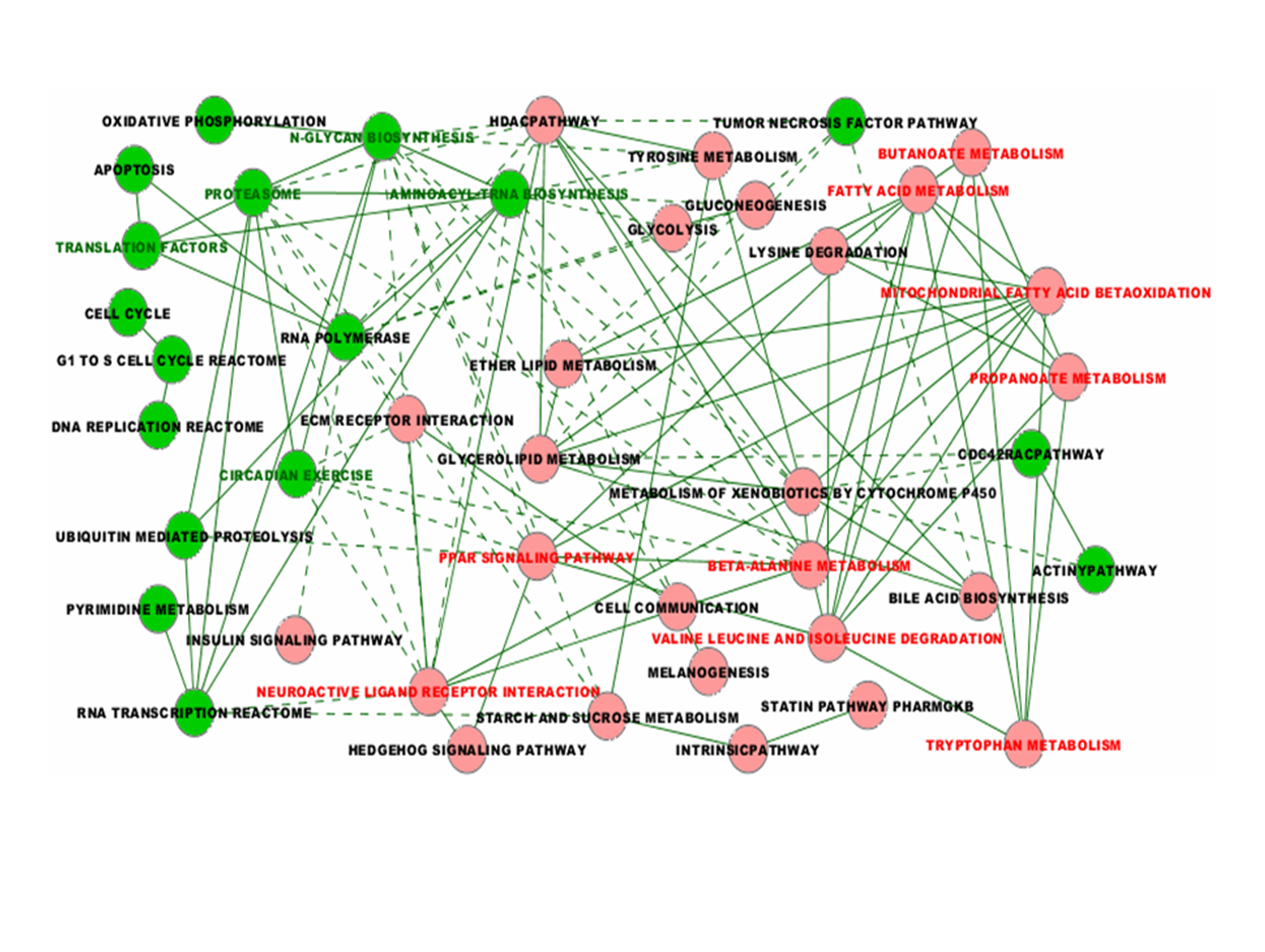

Supplement: Figure S1 — Network of molecular pathways showing anti-correlated behavior. Hubs are indicated with red and green fonts. Pink and green nodes represent pathways associated to Group A and B, respectively. (TIF) [file pone.0027819.s001.tif]
