# Supplementary material for: Existence of Inverted Profile in Chemically Responsive Molecular Pathways in the Zebrafish Liver
Source: PLoS One. 2011 Nov 29;6(11):e27819. doi: 10.1371/journal.pone.0027819 (PMC3226580; doi:10.1371/journal.pone.0027819)
Supplement: Table S1 — Categories, types, and experimental summary of hepatic transcriptomic data used in this study. As = Arsenic (V); BAP = Benzo-[a]-pyrene; CA = Chloroaniline; E2 = Estradiol; Hg = Mercury (II) chloride; NP = 4-Nitrophenol. (DOC) [file pone.0027819.s006.doc]

**Table S1.** Categories, types, and experimental summary of hepatic transcriptomic data used in this study. As = Arsenic (V); BAP = Benzo-[a]-pyrene; CA = Chloroaniline; E2 = Estradiol; Hg = Mercury (II) chloride; NP = 4-Nitrophenol

| **Category of perturbations** | **Sample Label** | **Treatment Conditions** | **GEO Series Accession** |
| --- | --- | --- | --- |
| **Physiological** | Liver Starved Female | Starvation, 21 days, female fish | GSE11107 |
|  | Female Liver Dietary | 25% carbohydrate diet, female fish | GSE8856 |
|  | Male Liver Dietary | 25% carbohydrate diet, male fish | GSE8856 |
| **Low or medium toxic levels,** | 5BAP 24h Male | 5 µg/L, 24 h, male fish | GSE30035 |
| **short exposure (8 and 24h)** | 50BAP 24h Male | 50 µg/L, 24 h, male fish | GSE30035 |
|  | 05E2 24h Male | 0.5 µg/L, 24h, male fish | GSE30050 |
|  | 5E2 24h Male | 5 µg/L, 24h, male fish | GSE30050 |
| **Low or medium toxic levels,** | 5BAP 96h Male | 5 µg/L, 96 h, male fish | GSE30035 |
| **long exposure (48 and 96h)** | 50BAP 96h Male | 50 µg/L, 96 h, male fish | GSE30035 |
|  | 05E2 96h Male | 0.5 µg/L, 96h, male fish | GSE30050 |
|  | 5E2 96h Male | 5 µg/L, 96h, male fish | GSE30050 |
| **High toxic levels,** | 500BAP 24h Male | 500 µg/L, 24 h, male fish | GSE30035 |
| **short exposure (8 and 24h)** | 50E2 24h Male | 50 µg/L, 24h, male fish | GSE30050 |
|  | NP 8h Female | 7 mg/L, 8h, female fish | GSE30060 |
|  | NP 24h Female | 7 mg/L, 24h, female fish | GSE30060 |
|  | NP 8h Male | 7 mg/L, 8h, male fish | GSE30058 |
|  | NP 24h Male | 7 mg/L, 24h, male fish | GSE30058 |
|  | CA 8h Female | 20 mg/L, 8h, female fish | GSE30057 |
|  | CA 24h Female | 20 mg/L, 24h, female fish | GSE30057 |
|  | CA 8h Male | 20 mg/L, 8h, male fish | GSE30055 |
|  | CA 24h Male | 20 mg/L, 24h, male fish | GSE30055 |
|  | Hg 8h Female | 200 µg/L, 8h, female fish | GSE18861 |
|  | Hg 24h Female | 200 µg/L, 24h, female fish | GSE18861 |
|  | As 8h Female | 15 ppm (~192 µM), 8h, female fish | GSE30062 |
|  | As 24h Female | 15 ppm (~192 µM), 24h, female fish | GSE30062 |
|  | As 8h Male | 15 ppm (~192 µM), 8h, male fish | GSE3048 |
|  | As 24h Male | 15 ppm (~192 µM), 24h, male fish | GSE3048 |
| **High toxic levels,** | 500BAP 96h Male | 500 µg/L, 96 h, male fish | GSE30035 |
| **long exposure (48 and 96h)** | 50E2 96h Male | 50 µg/L, 96h, male fish | GSE30050 |
|  | NP 48h Female | 7 mg/L, 48h, female fish | GSE30060 |
|  | NP 96h Female | 7 mg/L, 96h, female fish | GSE30060 |
|  | NP 48h Male | 7 mg/L, 48h, male fish | GSE30058 |
|  | NP 96h Male | 7 mg/L, 96h, male fish | GSE30058 |
|  | CA 48h Female | 20 mg/L, 48h, female fish | GSE30057 |
|  | CA 96h Female | 20 mg/L, 96h, female fish | GSE30057 |
|  | CA 48h Male | 20 mg/L, 48h, male fish | GSE30055 |
|  | CA 96h Male | 20 mg/L, 96h, male fish | GSE30055 |
|  | Hg 48h Female | 200 µg/L, 48h, female fish | GSE18861 |
|  | Hg 96h Female | 200 µg/L, 96h, female fish | GSE18861 |
|  | As 48h Female | 15 ppm (~192 µM), 48h, female fish | GSE30062 |
|  | As 96h Female | 15 ppm (~192 µM), 96h, female fish | GSE30062 |
|  | As 48h Male | 15 ppm (~192 µM), 48h, male fish | GSE3048 |
|  | As 96h Male | 15 ppm (~192 µM), 96h, male fish | GSE3048 |
