# Supplementary material for: Existence of Inverted Profile in Chemically Responsive Molecular Pathways in the Zebrafish Liver
Source: PLoS One. 2011 Nov 29;6(11):e27819. doi: 10.1371/journal.pone.0027819 (PMC3226580; doi:10.1371/journal.pone.0027819)
Supplement: Table S2 — Categories of pathways at different levels of responsiveness and degree of connectivities. HR = Highly responsive; MR = Medium responsive; LR = Low responsive; HC = Highly connected; LC = Low connected. (DOC) [file pone.0027819.s007.doc]

**Table S2.** Categories of pathways at different levels of responsiveness and degree of connectivities.

HR-HC (Hub): High responsive (>= 10 significant responses) and high degree of correlation connectivity (>= 10 connections)

| **GENESET** | **Number of significant responses (NP<0.05)** | **Degree of connectivity (Correlation > 0.5)** |
| --- | --- | --- |
| IL6PATHWAY | 10 | 14 |
| HSA04080_NEUROACTIVE_LIGAND_RECEPTOR_INTERACTION | 10 | 17 |
| MITOCHONDRIAL_FATTY_ACID_BETAOXIDATION | 11 | 13 |
| HSA00650_BUTANOATE_METABOLISM | 11 | 15 |
| TRANSLATION_FACTORS | 12 | 12 |
| HSA00640_PROPANOATE_METABOLISM | 12 | 16 |
| BETA_ALANINE_METABOLISM | 12 | 17 |
| HSA00380_TRYPTOPHAN_METABOLISM | 13 | 12 |
| CIRCADIAN_EXERCISE | 13 | 14 |
| VALINE_LEUCINE_AND_ISOLEUCINE_DEGRADATION | 13 | 17 |
| HSA00970_AMINOACYL_TRNA_BIOSYNTHESIS | 18 | 30 |
| HSA00510_N_GLYCAN_BIOSYNTHESIS | 21 | 22 |
| HSA00071_FATTY_ACID_METABOLISM | 22 | 19 |
| HSA03320_PPAR_SIGNALING_PATHWAY | 25 | 14 |
| HSA03010_RIBOSOME | 27 | 11 |
| HSA03050_PROTEASOME | 30 | 21 |

HR-LC: High responsive (>= 10 significant responses) and low degree of correlation connectivity (0 to 9 connections)

| **GENESET** | **Number of significant responses (NP<0.05)** | **Degree of connectivity (Correlation > 0.5)** |
| --- | --- | --- |
| NUCLEAR_RECEPTORS | 10 | 5 |
| HSA04110_CELL_CYCLE | 10 | 6 |
| HSA00561_GLYCEROLIPID_METABOLISM | 10 | 7 |
| PYRUVATE_METABOLISM | 10 | 7 |
| LYSINE_DEGRADATION | 10 | 9 |
| APOPTOSIS | 12 | 5 |
| HSA04610_COMPLEMENT_AND_COAGULATION_CASCADES | 22 | 6 |
| HSA01430_CELL_COMMUNICATION | 10 | 4 |
| HSA00190_OXIDATIVE_PHOSPHORYLATION | 27 | 3 |

MR-HC: Medium responsive (5 to 9 significant responses) and high degree of correlation connectivity (>= 10 connections)

| **GENESET** | **Number of significant responses (NP<0.05)** | **Degree of connectivity (Correlation > 0.5)** |
| --- | --- | --- |
| PENTOSE_PHOSPHATE_PATHWAY | 5 | 11 |
| RNA_TRANSCRIPTION_REACTOME | 5 | 12 |
| BIOPEPTIDESPATHWAY | 5 | 16 |
| HSA00980_METABOLISM_OF_XENOBIOTICS_BY_CYTOCHROME_P450 | 5 | 19 |
| HDACPATHWAY | 5 | 29 |
| INTRINSICPATHWAY | 6 | 11 |
| HSA04120_UBIQUITIN_MEDIATED_PROTEOLYSIS | 6 | 12 |
| METPATHWAY | 6 | 18 |
| GLYCEROLIPID_METABOLISM | 7 | 10 |
| HSA00790_FOLATE_BIOSYNTHESIS | 7 | 12 |
| PURINE_METABOLISM | 7 | 17 |
| GLUCONEOGENESIS | 8 | 11 |
| HSA00480_GLUTATHIONE_METABOLISM | 8 | 13 |
| GLYCOLYSIS | 9 | 10 |
| HSA00120_BILE_ACID_BIOSYNTHESIS | 9 | 11 |
| HSA00632_BENZOATE_DEGRADATION_VIA_COA_LIGATION | 9 | 12 |
| HSA03020_RNA_POLYMERASE | 9 | 36 |

MR-LC: Medium responsive (5 to 9 significant responses) and medium degree of correlation connectivity (0 to 9 connections)

| **GENESET** | **Number of significant responses (NP<0.05)** | **Degree of connectivity (Correlation > 0.5)** |
| --- | --- | --- |
| IGF1PATHWAY | 5 | 9 |
| HSA00271_METHIONINE_METABOLISM | 6 | 7 |
| G_PROTEIN_SIGNALING | 7 | 5 |
| HSA04512_ECM_RECEPTOR_INTERACTION | 7 | 5 |
| MRNA_PROCESSING_REACTOME | 7 | 6 |
| DNA_REPLICATION_REACTOME | 7 | 8 |
| HSA00100_BIOSYNTHESIS_OF_STEROIDS | 8 | 6 |
| HSA00240_PYRIMIDINE_METABOLISM | 8 | 6 |
| HSA04060_CYTOKINE_CYTOKINE_RECEPTOR_INTERACTION | 5 | 0 |
| HSA04520_ADHERENS_JUNCTION | 5 | 0 |
| CALCIUM_REGULATION_IN_CARDIAC_CELLS | 5 | 1 |
| HSA04612_ANTIGEN_PROCESSING_AND_PRESENTATION | 5 | 1 |
| HSA00252_ALANINE_AND_ASPARTATE_METABOLISM | 5 | 2 |
| HSA04510_FOCAL_ADHESION | 5 | 2 |
| ST_FAS_SIGNALING_PATHWAY | 5 | 3 |
| HSA00020_CITRATE_CYCLE | 5 | 4 |
| ALKPATHWAY | 6 | 0 |
| HSA04630_JAK_STAT_SIGNALING_PATHWAY | 6 | 0 |
| HSA04670_LEUKOCYTE_TRANSENDOTHELIAL_MIGRATION | 6 | 0 |
| HSA04340_HEDGEHOG_SIGNALING_PATHWAY | 6 | 2 |
| G1_TO_S_CELL_CYCLE_REACTOME | 6 | 4 |
| HSA04020_CALCIUM_SIGNALING_PATHWAY | 7 | 1 |
| HSA04514_CELL_ADHESION_MOLECULES | 7 | 1 |
| SMOOTH_MUSCLE_CONTRACTION | 7 | 1 |
| HSA04910_INSULIN_SIGNALING_PATHWAY | 7 | 2 |
| HSA04916_MELANOGENESIS | 8 | 3 |
| HSA04920_ADIPOCYTOKINE_SIGNALING_PATHWAY | 9 | 1 |
| NFATPATHWAY | 9 | 1 |
| HSA04530_TIGHT_JUNCTION | 9 | 2 |

LR-HC: Low responsive (0 to 4 significant responses) and high degree of correlation connectivity (>= 10 connections)

| **GENESET** | **Number of significant responses (NP<0.05)** | **Degree of connectivity (Correlation > 0.5)** |
| --- | --- | --- |
| CHEMICALPATHWAY | 0 | 11 |
| ECMPATHWAY | 0 | 21 |
| RHOPATHWAY | 1 | 10 |
| HSA00512_O_GLYCAN_BIOSYNTHESIS | 1 | 11 |
| RASPATHWAY | 1 | 12 |
| EGFPATHWAY | 2 | 11 |
| UBIQUINONE_BIOSYNTHESIS | 2 | 11 |
| EPOPATHWAY | 2 | 16 |
| PROSTAGLANDIN_SYNTHESIS_REGULATION | 2 | 21 |
| ST_ADRENERGIC | 2 | 24 |
| AT1RPATHWAY | 3 | 10 |
| EDG1PATHWAY | 3 | 10 |
| HSA03030_DNA_POLYMERASE | 3 | 10 |
| STARCH_AND_SUCROSE_METABOLISM | 3 | 12 |
| HSA04012_ERBB_SIGNALING_PATHWAY | 3 | 13 |
| PDGFPATHWAY | 3 | 15 |
| GLUTAMATE_METABOLISM | 3 | 17 |
| GPCRDB_CLASS_A_RHODOPSIN_LIKE | 3 | 24 |
| GLYCEROPHOSPHOLIPID_METABOLISM | 4 | 10 |
| SIG_IL4RECEPTOR_IN_B_LYPHOCYTES | 4 | 10 |
| STATIN_PATHWAY_PHARMGKB | 4 | 10 |
| ST_TUMOR_NECROSIS_FACTOR_PATHWAY | 4 | 12 |
| TYROSINE_METABOLISM | 4 | 18 |
| INTEGRINPATHWAY | 4 | 20 |

LR-LC: Low responsive (0 to 4 significant responses) and medium degree of correlation connectivity (0 to 9 connections)

| **GENESET** | **Number of significant responses (NP<0.05)** | **Degree of connectivity (Correlation > 0.5)** |
| --- | --- | --- |
| SIG_PIP3_SIGNALING_IN_CARDIAC_MYOCTES | 0 | 5 |
| UREA_CYCLE_AND_METABOLISM_OF_AMINO_GROUPS | 0 | 5 |
| KERATINOCYTEPATHWAY | 0 | 6 |
| CCR3PATHWAY | 0 | 8 |
| FCER1PATHWAY | 1 | 5 |
| ST_WNT_BETA_CATENIN_PATHWAY | 1 | 5 |
| GSK3PATHWAY | 1 | 6 |
| HSA00150_ANDROGEN_AND_ESTROGEN_METABOLISM | 1 | 6 |
| IGF1MTORPATHWAY | 1 | 6 |
| PYK2PATHWAY | 1 | 6 |
| HSA00562_INOSITOL_PHOSPHATE_METABOLISM | 1 | 7 |
| HSA02010_ABC_TRANSPORTERS_GENERAL | 1 | 7 |
| TPOPATHWAY | 1 | 7 |
| DEATHPATHWAY | 1 | 8 |
| HSA01032_GLYCAN_STRUCTURES_DEGRADATION | 1 | 8 |
| HSA04640_HEMATOPOIETIC_CELL_LINEAGE | 1 | 8 |
| NTHIPATHWAY | 1 | 8 |
| ST_GA13_PATHWAY | 1 | 8 |
| ATMPATHWAY | 1 | 9 |
| HSA04650_NATURAL_KILLER_CELL_MEDIATED_CYTOTOXICITY | 1 | 9 |
| HSA04664_FC_EPSILON_RI_SIGNALING_PATHWAY | 2 | 5 |
| MCALPAINPATHWAY | 2 | 5 |
| HSA04810_REGULATION_OF_ACTIN_CYTOSKELETON | 2 | 6 |
| SIG_CD40PATHWAYMAP | 2 | 6 |
| HSA04720_LONG_TERM_POTENTIATION | 2 | 7 |
| ST_B_CELL_ANTIGEN_RECEPTOR | 2 | 7 |
| SIG_INSULIN_RECEPTOR_PATHWAY_IN_CARDIAC_MYOCYTES | 2 | 8 |
| CDC42RACPATHWAY | 3 | 5 |
| HSA00450_SELENOAMINO_ACID_METABOLISM | 3 | 7 |
| HSA00565_ETHER_LIPID_METABOLISM | 3 | 7 |
| HSA04730_LONG_TERM_DEPRESSION | 3 | 7 |
| SIG_BCR_SIGNALING_PATHWAY | 3 | 9 |
| HSA04010_MAPK_SIGNALING_PATHWAY | 4 | 5 |
| TNFR1PATHWAY | 4 | 5 |
| ERKPATHWAY | 4 | 7 |
| ONE_CARBON_POOL_BY_FOLATE | 4 | 7 |
| STRIATED_MUSCLE_CONTRACTION | 4 | 7 |
| ACTINYPATHWAY | 4 | 8 |
| HSA04360_AXON_GUIDANCE | 0 | 0 |
| ST_PHOSPHOINOSITIDE_3_KINASE_PATHWAY | 0 | 0 |
| EIF4PATHWAY | 0 | 1 |
| ST_G_ALPHA_I_PATHWAY | 0 | 1 |
| ERK5PATHWAY | 0 | 2 |
| P38MAPKPATHWAY | 0 | 2 |
| PEPTIDE_GPCRS | 0 | 2 |
| ST_GAQ_PATHWAY | 0 | 2 |
| ETSPATHWAY | 0 | 3 |
| NGFPATHWAY | 0 | 3 |
| RACCYCDPATHWAY | 0 | 3 |
| ARAPPATHWAY | 0 | 4 |
| RARRXRPATHWAY | 0 | 4 |
| SIG_PIP3_SIGNALING_IN_B_LYMPHOCYTES | 0 | 4 |
| CARM_ERPATHWAY | 1 | 1 |
| G1PATHWAY | 1 | 2 |
| IL2PATHWAY | 1 | 2 |
| HSA00340_HISTIDINE_METABOLISM | 1 | 3 |
| MPRPATHWAY | 1 | 3 |
| STRESSPATHWAY | 1 | 3 |
| HSA00590_ARACHIDONIC_ACID_METABOLISM | 1 | 4 |
| HSA04660_T_CELL_RECEPTOR_SIGNALING_PATHWAY | 1 | 4 |
| P53HYPOXIAPATHWAY | 1 | 4 |
| TCRPATHWAY | 1 | 4 |
| GPCRPATHWAY | 2 | 0 |
| HSA04150_MTOR_SIGNALING_PATHWAY | 2 | 0 |
| HSA04620_TOLL_LIKE_RECEPTOR_SIGNALING_PATHWAY | 2 | 0 |
| ST_JNK_MAPK_PATHWAY | 2 | 0 |
| HSA04320_DORSO_VENTRAL_AXIS_FORMATION | 2 | 1 |
| PHOSPHATIDYLINOSITOL_SIGNALING_SYSTEM | 2 | 1 |
| HSA04540_GAP_JUNCTION | 2 | 2 |
| SIG_REGULATION_OF_THE_ACTIN_CYTOSKELETON_BY_RHO_GTPASES | 2 | 2 |
| FRUCTOSE_AND_MANNOSE_METABOLISM | 2 | 3 |
| PROSTAGLANDIN_AND_LEUKOTRIENE_METABOLISM | 2 | 3 |
| HSA00670_ONE_CARBON_POOL_BY_FOLATE | 2 | 4 |
| HSA00530_AMINOSUGARS_METABOLISM | 3 | 0 |
| HSA04350_TGF_BETA_SIGNALING_PATHWAY | 3 | 0 |
| ARGININE_AND_PROLINE_METABOLISM | 3 | 1 |
| IL2RBPATHWAY | 3 | 1 |
| CERAMIDEPATHWAY | 3 | 3 |
| CREBPATHWAY | 3 | 3 |
| TYPE_III_SECRETION_SYSTEM | 3 | 4 |
| HSA04330_NOTCH_SIGNALING_PATHWAY | 4 | 0 |
| CALCINEURIN_NF_AT_SIGNALING | 4 | 1 |
| CXCR4PATHWAY | 4 | 1 |
| GLYCINE_SERINE_AND_THREONINE_METABOLISM | 4 | 1 |
| HSA04912_GNRH_SIGNALING_PATHWAY | 4 | 1 |
| VEGFPATHWAY | 4 | 1 |
| HSA03022_BASAL_TRANSCRIPTION_FACTORS | 4 | 3 |
| HSA04115_P53_SIGNALING_PATHWAY | 4 | 3 |
| HSA04310_WNT_SIGNALING_PATHWAY | 4 | 3 |
| SPPAPATHWAY | 4 | 3 |
| ATP_SYNTHESIS | 4 | 4 |
| FMLPPATHWAY | 4 | 4 |
