# Supplementary material for: Existence of Inverted Profile in Chemically Responsive Molecular Pathways in the Zebrafish Liver
Source: PLoS One. 2011 Nov 29;6(11):e27819. doi: 10.1371/journal.pone.0027819 (PMC3226580; doi:10.1371/journal.pone.0027819)
Supplement: Table S3 — Categories of pathways with known associated functions showing significant correlation coefficients. (DOC) [file pone.0027819.s008.doc]

**Table S3.** Categories of pathways with known associated functions showing significant correlation coefficients.

Glucose and energetic metabolism

| **Pathway A** | **Pathway B** | **Correlation Coefficient** | **P-value** |
| --- | --- | --- | --- |
| GLYCOLYSIS | GLUCONEOGENESIS | 0.99991 | 2.28E-78 |
| PENTOSE_PHOSPHATE_PATHWAY | HSA00562_INOSITOL_PHOSPHATE_METABOLISM | 0.70626 | 1.21E-07 |
| PYRUVATE_METABOLISM | HSA00640_PROPANOATE_METABOLISM | 0.56841 | 7.00E-05 |
| PYRUVATE_METABOLISM | GLUCONEOGENESIS | 0.56212 | 8.76E-05 |
| PYRUVATE_METABOLISM | GLYCOLYSIS | 0.55833 | 0.00010001 |
| STARCH_AND_SUCROSE_METABOLISM | PENTOSE_PHOSPHATE_PATHWAY | 0.55571 | 0.00010951 |
| HSA00020_CITRATE_CYCLE | GLUCONEOGENESIS | 0.55129 | 0.00012742 |
| HSA00020_CITRATE_CYCLE | GLYCOLYSIS | 0.55004 | 0.00013296 |

Fatty acid metabolism

| **Pathway A** | **Pathway B** | **Correlation Coefficient** | **P-value** |
| --- | --- | --- | --- |
| VALINE_LEUCINE_AND_ISOLEUCINE_DEGRADATION | HSA00071_FATTY_ACID_METABOLISM | 0.86583 | 6.64E-14 |
| HSA00071_FATTY_ACID_METABOLISM | BETA_ALANINE_METABOLISM | 0.75448 | 5.13E-09 |
| HSA03320_PPAR_SIGNALING_PATHWAY | BETA_ALANINE_METABOLISM | 0.74645 | 9.11E-09 |
| HSA00561_GLYCEROLIPID_METABOLISM | HSA00071_FATTY_ACID_METABOLISM | 0.71685 | 6.41E-08 |
| HSA03320_PPAR_SIGNALING_PATHWAY | HSA00071_FATTY_ACID_METABOLISM | 0.68619 | 3.79E-07 |
| VALINE_LEUCINE_AND_ISOLEUCINE_DEGRADATION | HSA00650_BUTANOATE_METABOLISM | 0.65546 | 1.84E-06 |
| HSA03320_PPAR_SIGNALING_PATHWAY | HSA00561_GLYCEROLIPID_METABOLISM | 0.64833 | 2.59E-06 |
| HSA00650_BUTANOATE_METABOLISM | HSA00071_FATTY_ACID_METABOLISM | 0.64538 | 2.97E-06 |
| HSA00650_BUTANOATE_METABOLISM | HSA00632_BENZOATE_DEGRADATION_VIA_COA_LIGATION | 0.63094 | 5.74E-06 |
| MITOCHONDRIAL_FATTY_ACID_BETAOXIDATION | HSA00565_ETHER_LIPID_METABOLISM | 0.62441 | 7.65E-06 |
| HSA00100_BIOSYNTHESIS_OF_STEROIDS | GLYCEROPHOSPHOLIPID_METABOLISM | 0.6141 | 1.19E-05 |
| HSA00640_PROPANOATE_METABOLISM | HSA00071_FATTY_ACID_METABOLISM | 0.59751 | 2.33E-05 |
| MITOCHONDRIAL_FATTY_ACID_BETAOXIDATION | HSA00071_FATTY_ACID_METABOLISM | 0.58553 | 3.71E-05 |
| HSA00640_PROPANOATE_METABOLISM | HSA00632_BENZOATE_DEGRADATION_VIA_COA_LIGATION | 0.5726 | 6.01E-05 |
| PROSTAGLANDIN_SYNTHESIS_REGULATION | HSA00120_BILE_ACID_BIOSYNTHESIS | 0.56866 | 6.94E-05 |
| HSA00565_ETHER_LIPID_METABOLISM | HSA00071_FATTY_ACID_METABOLISM | 0.5608 | 9.17E-05 |
| MITOCHONDRIAL_FATTY_ACID_BETAOXIDATION | BETA_ALANINE_METABOLISM | 0.55977 | 9.51E-05 |
| MITOCHONDRIAL_FATTY_ACID_BETAOXIDATION | HSA03320_PPAR_SIGNALING_PATHWAY | 0.54318 | 0.00016736 |
| HSA00565_ETHER_LIPID_METABOLISM | HSA00561_GLYCEROLIPID_METABOLISM | 0.51773 | 0.00037711 |
| HSA00632_BENZOATE_DEGRADATION_VIA_COA_LIGATION | HSA00071_FATTY_ACID_METABOLISM | 0.50365 | 0.00057548 |
| VALINE_LEUCINE_AND_ISOLEUCINE_DEGRADATION | MITOCHONDRIAL_FATTY_ACID_BETAOXIDATION | 0.50269 | 0.00059179 |

Amino acid metabolism

| **Pathway A** | **Pathway B** | **Correlation Coefficient** | **P-value** |
| --- | --- | --- | --- |
| HSA00380_TRYPTOPHAN_METABOLISM | HSA00071_FATTY_ACID_METABOLISM | 0.69817 | 1.94E-07 |
| HSA00650_BUTANOATE_METABOLISM | HSA00380_TRYPTOPHAN_METABOLISM | 0.69722 | 2.05E-07 |
| HSA00632_BENZOATE_DEGRADATION_VIA_COA_LIGATION | HSA00380_TRYPTOPHAN_METABOLISM | 0.68953 | 3.16E-07 |
| VALINE_LEUCINE_AND_ISOLEUCINE_DEGRADATION | LYSINE_DEGRADATION | 0.66148 | 1.37E-06 |
| VALINE_LEUCINE_AND_ISOLEUCINE_DEGRADATION | BETA_ALANINE_METABOLISM | 0.64478 | 3.06E-06 |
| HSA00561_GLYCEROLIPID_METABOLISM | BETA_ALANINE_METABOLISM | 0.59899 | 2.20E-05 |
| VALINE_LEUCINE_AND_ISOLEUCINE_DEGRADATION | HSA00380_TRYPTOPHAN_METABOLISM | 0.59345 | 2.74E-05 |
| HSA00640_PROPANOATE_METABOLISM | HSA00380_TRYPTOPHAN_METABOLISM | 0.51238 | 0.00044376 |

Cell cycle and DNA replication

| **Pathway A** | **Pathway B** | **Correlation Coefficient** | **P-value** |
| --- | --- | --- | --- |
| HSA04110_CELL_CYCLE | G1_TO_S_CELL_CYCLE_REACTOME | 0.66126 | 1.39E-06 |
| DNA_REPLICATION_REACTOME | ATMPATHWAY | 0.6448 | 3.06E-06 |
| G1_TO_S_CELL_CYCLE_REACTOME | DNA_REPLICATION_REACTOME | 0.60776 | 1.54E-05 |
| HSA04110_CELL_CYCLE | DNA_REPLICATION_REACTOME | 0.59964 | 2.14E-05 |
| HSA04110_CELL_CYCLE | G1PATHWAY | 0.59335 | 2.75E-05 |
| HSA03030_DNA_POLYMERASE | ATMPATHWAY | 0.58993 | 3.14E-05 |
| HSA03030_DNA_POLYMERASE | DNA_REPLICATION_REACTOME | 0.57313 | 5.90E-05 |
| HSA03030_DNA_POLYMERASE | HSA03020_RNA_POLYMERASE | 0.55916 | 9.71E-05 |
| HSA04110_CELL_CYCLE | ATMPATHWAY | 0.55855 | 9.92E-05 |

Receptor-mediated signal transduction

| **Pathway A** | **Pathway B** | **Correlation Coefficient** | **P-value** |
| --- | --- | --- | --- |
| PDGFPATHWAY | EGFPATHWAY | 0.88726 | 2.32E-15 |
| IL6PATHWAY | EGFPATHWAY | 0.74444 | 1.05E-08 |
| METPATHWAY | EGFPATHWAY | 0.71194 | 8.65E-08 |
| PDGFPATHWAY | METPATHWAY | 0.70536 | 1.28E-07 |
| G_PROTEIN_SIGNALING | CALCIUM_REGULATION_IN_CARDIAC_CELLS | 0.68179 | 4.81E-07 |
| PDGFPATHWAY | IGF1PATHWAY | 0.66564 | 1.11E-06 |
| ST_WNT_BETA_CATENIN_PATHWAY | SIG_INSULIN_RECEPTOR_PATHWAY_IN_CARDIAC_MYOCYTES | 0.63709 | 4.36E-06 |
| GSK3PATHWAY | CREBPATHWAY | 0.63546 | 4.69E-06 |
| IGF1PATHWAY | EGFPATHWAY | 0.59061 | 3.06E-05 |
| PDGFPATHWAY | ERKPATHWAY | 0.56495 | 7.92E-05 |
| PDGFPATHWAY | HSA04012_ERBB_SIGNALING_PATHWAY | 0.56153 | 8.94E-05 |
| METPATHWAY | HSA04012_ERBB_SIGNALING_PATHWAY | 0.5542 | 0.00011536 |
| PYK2PATHWAY | METPATHWAY | 0.54258 | 0.00017074 |
| METPATHWAY | ERKPATHWAY | 0.53199 | 0.00024115 |
| PYK2PATHWAY | ERKPATHWAY | 0.5281 | 0.00027293 |
| SIG_INSULIN_RECEPTOR_PATHWAY_IN_CARDIAC_MYOCYTES | HSA04012_ERBB_SIGNALING_PATHWAY | 0.52805 | 0.00027337 |
| SIG_PIP3_SIGNALING_IN_CARDIAC_MYOCTES | METPATHWAY | 0.52299 | 0.00032054 |
| SIG_PIP3_SIGNALING_IN_CARDIAC_MYOCTES | HSA04012_ERBB_SIGNALING_PATHWAY | 0.50491 | 0.00055449 |
| PHOSPHATIDYLINOSITOL_SIGNALING_SYSTEM | HSA04010_MAPK_SIGNALING_PATHWAY | 0.5004 | 0.00063279 |

Cytoskeleton and cell-cell interactions

| **Pathway A** | **Pathway B** | **Correlation Coefficient** | **P-value** |
| --- | --- | --- | --- |
| CDC42RACPATHWAY | ACTINYPATHWAY | 0.89858 | 2.95E-16 |
| METPATHWAY | INTEGRINPATHWAY | 0.82546 | 9.74E-12 |
| RASPATHWAY | ACTINYPATHWAY | 0.73591 | 1.88E-08 |
| RASPATHWAY | CDC42RACPATHWAY | 0.72981 | 2.81E-08 |
| INTEGRINPATHWAY | EGFPATHWAY | 0.66118 | 1.39E-06 |
| PDGFPATHWAY | INTEGRINPATHWAY | 0.6452 | 3.00E-06 |
| HSA04512_ECM_RECEPTOR_INTERACTION | HSA01430_CELL_COMMUNICATION | 0.63103 | 5.72E-06 |
| INTEGRINPATHWAY | IGF1PATHWAY | 0.58224 | 4.21E-05 |
| HSA04810_REGULATION_OF_ACTIN_CYTOSKELETON | AT1RPATHWAY | 0.57231 | 6.08E-05 |
| HSA04512_ECM_RECEPTOR_INTERACTION | HSA04080_NEUROACTIVE_LIGAND_RECEPTOR_INTERACTION | 0.55893 | 9.79E-05 |
| KERATINOCYTEPATHWAY | INTEGRINPATHWAY | 0.5474 | 0.00014538 |
| RHOPATHWAY | ECMPATHWAY | 0.53468 | 0.00022112 |
| HSA04810_REGULATION_OF_ACTIN_CYTOSKELETON | FMLPPATHWAY | 0.5262 | 0.00028978 |
| INTEGRINPATHWAY | ERKPATHWAY | 0.5201 | 0.00035053 |
| HSA04510_FOCAL_ADHESION | HSA04012_ERBB_SIGNALING_PATHWAY | 0.51545 | 0.00040435 |
| SMOOTH_MUSCLE_CONTRACTION | HSA04530_TIGHT_JUNCTION | 0.50995 | 0.00047736 |
| KERATINOCYTEPATHWAY | HSA04810_REGULATION_OF_ACTIN_CYTOSKELETON | 0.50816 | 0.00050351 |
| INTEGRINPATHWAY | HSA04012_ERBB_SIGNALING_PATHWAY | 0.50627 | 0.00053265 |

Stress, apoptosis, cell death and proteolysis

| **Pathway A** | **Pathway B** | **Correlation Coefficient** | **P-value** |
| --- | --- | --- | --- |
| HSA04120_UBIQUITIN_MEDIATED_PROTEOLYSIS | HSA03050_PROTEASOME | 0.65398 | 1.98E-06 |
| TNFR1PATHWAY | APOPTOSIS | 0.57879 | 4.79E-05 |
| ST_TUMOR_NECROSIS_FACTOR_PATHWAY | DEATHPATHWAY | 0.57711 | 5.10E-05 |
| P53HYPOXIAPATHWAY | INTRINSICPATHWAY | 0.50063 | 0.00062858 |
| STRESSPATHWAY | HSA04320_DORSO_VENTRAL_AXIS_FORMATION | -0.55494 | 0.00011246 |
| INTRINSICPATHWAY | HSA00670_ONE_CARBON_POOL_BY_FOLATE | -0.59283 | 2.80E-05 |

Immune responses

| **Pathway A** | **Pathway B** | **Correlation Coefficient** | **P-value** |
| --- | --- | --- | --- |
| SIG_IL4RECEPTOR_IN_B_LYMPHOCYTES | SIG_BCR_SIGNALING_PATHWAY | 0.6882 | 3.40E-07 |
| TCRPATHWAY | FCER1PATHWAY | 0.64045 | 3.74E-06 |
| IL6PATHWAY | HSA04660_T_CELL_RECEPTOR_SIGNALING_PATHWAY | 0.60062 | 2.06E-05 |
| SIG_BCR_SIGNALING_PATHWAY | HSA04012_ERBB_SIGNALING_PATHWAY | 0.59404 | 2.67E-05 |
| IL2PATHWAY | FCER1PATHWAY | 0.56933 | 6.77E-05 |
| HSA04650_NATURAL_KILLER_CELL_MEDIATED_CYTOTOXICITY | HSA04012_ERBB_SIGNALING_PATHWAY | 0.55265 | 0.00012164 |
| SIG_BCR_SIGNALING_PATHWAY | EGFPATHWAY | 0.54977 | 0.00013419 |
| ST_B_CELL_ANTIGEN_RECEPTOR | HSA04012_ERBB_SIGNALING_PATHWAY | 0.53518 | 0.00021761 |
| HSA04664_FC_EPSILON_RI_SIGNALING_PATHWAY | HSA04650_NATURAL_KILLER_CELL_MEDIATED_CYTOTOXICITY | 0.52242 | 0.00032618 |
| SIG_BCR_SIGNALING_PATHWAY | METPATHWAY | 0.52231 | 0.0003273 |
| HSA04664_FC_EPSILON_RI_SIGNALING_PATHWAY | HSA04012_ERBB_SIGNALING_PATHWAY | 0.5202 | 0.00034953 |
| SIG_IL4RECEPTOR_IN_B_LYMPHOCYTES | METPATHWAY | 0.51805 | 0.00037344 |
| SIG_INSULIN_RECEPTOR_PATHWAY_IN_CARDIAC_MYOCYTES | IL2RBPATHWAY | 0.51746 | 0.00038022 |
| IL6PATHWAY | IL2PATHWAY | 0.51216 | 0.00044671 |
| SIG_BCR_SIGNALING_PATHWAY | IGF1PATHWAY | 0.50144 | 0.00061382 |

Xenobiotics and epigenetic responses

| **Pathway A** | **Pathway B** | **Correlation Coefficient** | **P-value** |
| --- | --- | --- | --- |
| HSA00980_METABOLISM_OF_XENOBIOTICS_BY_CYTOCHROME_P450 | HDACPATHWAY | 0.77001 | 1.58E-09 |
| HSA00120_BILE_ACID_BIOSYNTHESIS | HDACPATHWAY | 0.64184 | 3.51E-06 |
| HSA00980_METABOLISM_OF_XENOBIOTICS_BY_CYTOCHROME_P450 | HSA00120_BILE_ACID_BIOSYNTHESIS | 0.63847 | 4.09E-06 |
| HSA03050_PROTEASOME | HDACPATHWAY | -0.57477 | 5.55E-05 |

Transcription, protein synthesis, and circadian clock regulation

| **Pathway A** | **Pathway B** | **Correlation Coefficient** | **P-value** |
| --- | --- | --- | --- |
| RNA_TRANSCRIPTION_REACTOME | HSA00240_PYRIMIDINE_METABOLISM | 0.71926 | 5.52E-08 |
| TRANSLATION_FACTORS | GLUTAMATE_METABOLISM | 0.71534 | 7.03E-08 |
| TRANSLATION_FACTORS | HSA03020_RNA_POLYMERASE | 0.68469 | 4.11E-07 |
| HSA03020_RNA_POLYMERASE | GLUTAMATE_METABOLISM | 0.66845 | 9.66E-07 |
| HSA00970_AMINOACYL_TRNA_BIOSYNTHESIS | GLUTAMATE_METABOLISM | 0.66671 | 1.05E-06 |
| RNA_TRANSCRIPTION_REACTOME | MRNA_PROCESSING_REACTOME | 0.60357 | 1.83E-05 |
| HSA03020_RNA_POLYMERASE | HSA00970_AMINOACYL_TRNA_BIOSYNTHESIS | 0.59075 | 3.04E-05 |
| TRANSLATION_FACTORS | HSA00970_AMINOACYL_TRNA_BIOSYNTHESIS | 0.57901 | 4.75E-05 |
| TRANSLATION_FACTORS | HSA00450_SELENOAMINO_ACID_METABOLISM | 0.5665 | 7.49E-05 |
| MRNA_PROCESSING_REACTOME | HSA00240_PYRIMIDINE_METABOLISM | 0.5372 | 0.00020377 |
| HSA03020_RNA_POLYMERASE | HDACPATHWAY | -0.57874 | 4.80E-05 |
| HSA03050_PROTEASOME | CIRCADIAN_EXERCISE | 0.63031 | 5.91E-06 |
