# Supplementary material for: Existence of Inverted Profile in Chemically Responsive Molecular Pathways in the Zebrafish Liver
Source: PLoS One. 2011 Nov 29;6(11):e27819. doi: 10.1371/journal.pone.0027819 (PMC3226580; doi:10.1371/journal.pone.0027819)
Supplement: Table S4 — Assigned anti-correlation scores to pathways showing significant responds to 20 selected conditions with obvious inverted activities of hubs as “seed”. Red and green fonts are hub pathways of Group A and B, respectively. Pink and green backgrounds are anti-correlated pathways assigned to Group A and B, respectively. See Materials and Methods for detail description for anti-correlation scores. (DOC) [file pone.0027819.s009.doc]

**Table S4.** Assigned anti-correlation scores to pathways showing significant responds to 20 selected conditions with obvious inverted activities of hubs as “seed”. Red and green fonts are hub pathways of Group A and B, respectively. Pink and green backgrounds are anti-correlated pathways assigned to Group A and B, respectively. See Materials and Methods for detail description for anti-correlation scores.

| **GENESET** | **A-Score** | **B-Score** | **Anti-Correlated Score** | **Assigned Anti-Correlated Group** | **Pathway Category** |
| --- | --- | --- | --- | --- | --- |
| **HSA00071_FATTY_ACID_METABOLISM** | 16 | -16 | 1 | **A** | HR-HC |
| **HSA00640_PROPANOATE_METABOLISM** | 10 | -10 | 1 | **A** | HR-HC |
| **MITOCHONDRIAL_FATTY_ACID_BETAOXIDATION** | 10 | -10 | 1 | **A** | HR-HC |
| **VALINE_LEUCINE_AND_ISOLEUCINE_DEGRADATION** | 9 | -9 | 1 | **A** | HR-HC |
| **BETA_ALANINE_METABOLISM** | 9 | -9 | 1 | **A** | HR-HC |
| **HSA00650_BUTANOATE_METABOLISM** | 9 | -9 | 1 | **A** | HR-HC |
| **HSA04080_NEUROACTIVE_LIGAND_RECEPTOR_INTERACTION** | 6 | -6 | 1 | **A** | HR-HC |
| **HSA00380_TRYPTOPHAN_METABOLISM** | 6 | -6 | 1 | **A** | HR-HC |
| **HSA03320_PPAR_SIGNALING_PATHWAY** | 16 | -16 | 0.88888889 | **A** | HR-HC |
| LYSINE_DEGRADATION | 8 | -8 | 1 | A | HR-LC |
| HSA00120_BILE_ACID_BIOSYNTHESIS | 7 | -7 | 1 | A | MR-HC |
| GLYCEROLIPID_METABOLISM | 6 | -6 | 1 | A | MR-HC |
| HSA01430_CELL_COMMUNICATION | 6 | -6 | 1 | A | HR-LC |
| HSA00980_METABOLISM_OF_XENOBIOTICS_BY_CYTOCHROME_P450 | 5 | -5 | 1 | A | MR-HC |
| GLUCONEOGENESIS | 5 | -5 | 1 | A | MR-HC |
| INTRINSICPATHWAY | 5 | -5 | 1 | A | MR-HC |
| GLYCOLYSIS | 5 | -5 | 1 | A | MR-HC |
| NFATPATHWAY | 5 | -5 | 1 | A | MR-LC |
| TYROSINE_METABOLISM | 4 | -4 | 1 | A | LR-HC |
| STATIN_PATHWAY_PHARMGKB | 4 | -4 | 1 | A | LR-HC |
| HSA04512_ECM_RECEPTOR_INTERACTION | 4 | -4 | 1 | A | MR-LC |
| HSA04916_MELANOGENESIS | 4 | -4 | 1 | A | MR-LC |
| HSA04910_INSULIN_SIGNALING_PATHWAY | 4 | -4 | 1 | A | MR-LC |
| HSA04340_HEDGEHOG_SIGNALING_PATHWAY | 4 | -4 | 1 | A | MR-LC |
| HSA04020_CALCIUM_SIGNALING_PATHWAY | 4 | -4 | 1 | A | MR-LC |
| HSA04514_CELL_ADHESION_MOLECULES | 4 | -4 | 1 | A | MR-LC |
| HDACPATHWAY | 3 | -3 | 1 | A | MR-HC |
| STARCH_AND_SUCROSE_METABOLISM | 3 | -3 | 1 | A | LR-HC |
| HSA00565_ETHER_LIPID_METABOLISM | 3 | -3 | 1 | A | LR-LC |
| ALKPATHWAY | 4 | -4 | 1 | A | MR-LC |
| **HSA00970_AMINOACYL_TRNA_BIOSYNTHESIS** | -12 | 12 | 1 | **B** | HR-HC |
| **HSA00510_N_GLYCAN_BIOSYNTHESIS** | -14 | 14 | 1 | **B** | HR-HC |
| **HSA03050_PROTEASOME** | -17 | 17 | 0.94444444 | **B** | HR-HC |
| **CIRCADIAN_EXERCISE** | -9 | 9 | 1 | **B** | HR-HC |
| **TRANSLATION_FACTORS** | -7 | 7 | 0.77777778 | **B** | HR-HC |
| HSA04120_UBIQUITIN_MEDIATED_PROTEOLYSIS | -3 | 3 | 1 | B | MR-HC |
| RNA_TRANSCRIPTION_REACTOME | -3 | 3 | 1 | B | MR-HC |
| ST_TUMOR_NECROSIS_FACTOR_PATHWAY | -3 | 3 | 1 | B | LR-HC |
| CDC42RACPATHWAY | -3 | 3 | 1 | B | LR-LC |
| G1_TO_S_CELL_CYCLE_REACTOME | -3 | 3 | 1 | B | MR-LC |
| CALCINEURIN_NF_AT_SIGNALING | -3 | 3 | 1 | B | LR-LC |
| DNA_REPLICATION_REACTOME | -4 | 4 | 1 | B | MR-LC |
| ACTINYPATHWAY | -4 | 4 | 1 | B | LR-LC |
| SMOOTH_MUSCLE_CONTRACTION | -4 | 4 | 1 | B | MR-LC |
| HSA00240_PYRIMIDINE_METABOLISM | -5 | 5 | 1 | B | MR-LC |
| HSA03020_RNA_POLYMERASE | -6 | 6 | 0.75 | B | MR-HC |
| HSA04110_CELL_CYCLE | -7 | 7 | 1 | B | HR-LC |
| APOPTOSIS | -8 | 8 | 1 | B | HR-LC |
| HSA00190_OXIDATIVE_PHOSPHORYLATION | -10 | 10 | 0.71428571 | B | HR-LC |
| HSA00632_BENZOATE_DEGRADATION_VIA_COA_LIGATION | 4 | -4 | 0.66666667 | NA | MR-HC |
| PYRUVATE_METABOLISM | 4 | -4 | 0.66666667 | NA | HR-LC |
| HSA04920_ADIPOCYTOKINE_SIGNALING_PATHWAY | 4 | -4 | 0.66666667 | NA | MR-LC |
| PENTOSE_PHOSPHATE_PATHWAY | 3 | -3 | 0.6 | NA | MR-HC |
| ONE_CARBON_POOL_BY_FOLATE | -3 | 3 | 0.6 | NA | LR-LC |
| NUCLEAR_RECEPTORS | 3 | -3 | 0.6 | NA | HR-LC |
| PURINE_METABOLISM | -2 | 2 | 0.5 | NA | MR-HC |
| HSA00790_FOLATE_BIOSYNTHESIS | -2 | 2 | 0.5 | NA | MR-HC |
| IGF1PATHWAY | 2 | -2 | 0.5 | NA | MR-LC |
| HSA04610_COMPLEMENT_AND_COAGULATION_CASCADES | 5 | -5 | 0.38461538 | NA | HR-LC |
| METPATHWAY | -1 | 1 | 0.33333333 | NA | MR-HC |
| BIOPEPTIDESPATHWAY | -1 | 1 | 0.33333333 | NA | MR-HC |
| **IL6PATHWAY** | 2 | -2 | 0.33333333 | NA | HR-HC |
| G_PROTEIN_SIGNALING | 1 | -1 | 0.33333333 | NA | MR-LC |
| HSA04530_TIGHT_JUNCTION | 1 | -1 | 0.33333333 | NA | MR-LC |
| HSA00252_ALANINE_AND_ASPARTATE_METABOLISM | 1 | -1 | 0.33333333 | NA | MR-LC |
| HSA04612_ANTIGEN_PROCESSING_AND_PRESENTATION | 1 | -1 | 0.33333333 | NA | MR-LC |
| GLYCINE_SERINE_AND_THREONINE_METABOLISM | -1 | 1 | 0.33333333 | NA | LR-LC |
| HSA04670_LEUKOCYTE_TRANSENDOTHELIAL_MIGRATION | 1 | -1 | 0.33333333 | NA | MR-LC |
| HSA04520_ADHERENS_JUNCTION | -1 | 1 | 0.33333333 | NA | MR-LC |
| **HSA03010_RIBOSOME** | 4 | -4 | 0.25 | NA | HR-HC |
| HSA00100_BIOSYNTHESIS_OF_STEROIDS | 1 | -1 | 0.2 | NA | MR-LC |
| GPCRDB_CLASS_A_RHODOPSIN_LIKE | 2 | -2 | NA | NA | LR-HC |
| ST_ADRENERGIC | 1 | -1 | NA | NA | LR-HC |
| PROSTAGLANDIN_SYNTHESIS_REGULATION | 2 | -2 | NA | NA | LR-HC |
| ECMPATHWAY | 0 | 0 | NA | NA | LR-HC |
| INTEGRINPATHWAY | -1 | 1 | NA | NA | LR-HC |
| GLUTAMATE_METABOLISM | 0 | 0 | NA | NA | LR-HC |
| EPOPATHWAY | -1 | 1 | NA | NA | LR-HC |
| PDGFPATHWAY | -1 | 1 | NA | NA | LR-HC |
| HSA00480_GLUTATHIONE_METABOLISM | 0 | 0 | NA | NA | MR-HC |
| HSA04012_ERBB_SIGNALING_PATHWAY | 0 | 0 | NA | NA | LR-HC |
| RASPATHWAY | -1 | 1 | NA | NA | LR-HC |
| EGFPATHWAY | 0 | 0 | NA | NA | LR-HC |
| UBIQUINONE_BIOSYNTHESIS | -2 | 2 | NA | NA | LR-HC |
| HSA00512_O_GLYCAN_BIOSYNTHESIS | 0 | 0 | NA | NA | LR-HC |
| CHEMICALPATHWAY | 0 | 0 | NA | NA | LR-HC |
| GLYCEROPHOSPHOLIPID_METABOLISM | 1 | -1 | NA | NA | LR-HC |
| SIG_IL4RECEPTOR_IN_B_LYPHOCYTES | 0 | 0 | NA | NA | LR-HC |
| AT1RPATHWAY | -1 | 1 | NA | NA | LR-HC |
| EDG1PATHWAY | 0 | 0 | NA | NA | LR-HC |
| HSA03030_DNA_POLYMERASE | -2 | 2 | NA | NA | LR-HC |
| RHOPATHWAY | -1 | 1 | NA | NA | LR-HC |
| SIG_BCR_SIGNALING_PATHWAY | -1 | 1 | NA | NA | LR-LC |
| ATMPATHWAY | 0 | 0 | NA | NA | LR-LC |
| HSA04650_NATURAL_KILLER_CELL_MEDIATED_CYTOTOXICITY | 0 | 0 | NA | NA | LR-LC |
| SIG_INSULIN_RECEPTOR_PATHWAY_IN_CARDIAC_MYOCYTES | 1 | -1 | NA | NA | LR-LC |
| DEATHPATHWAY | -1 | 1 | NA | NA | LR-LC |
| HSA01032_GLYCAN_STRUCTURES_DEGRADATION | 1 | -1 | NA | NA | LR-LC |
| HSA04640_HEMATOPOIETIC_CELL_LINEAGE | 0 | 0 | NA | NA | LR-LC |
| NTHIPATHWAY | -1 | 1 | NA | NA | LR-LC |
| ST_GA13_PATHWAY | 0 | 0 | NA | NA | LR-LC |
| CCR3PATHWAY | 0 | 0 | NA | NA | LR-LC |
| HSA00271_METHIONINE_METABOLISM | 0 | 0 | NA | NA | MR-LC |
| ERKPATHWAY | -1 | 1 | NA | NA | LR-LC |
| STRIATED_MUSCLE_CONTRACTION | 0 | 0 | NA | NA | LR-LC |
| HSA00450_SELENOAMINO_ACID_METABOLISM | -1 | 1 | NA | NA | LR-LC |
| HSA04730_LONG_TERM_DEPRESSION | 0 | 0 | NA | NA | LR-LC |
| HSA04720_LONG_TERM_POTENTIATION | 0 | 0 | NA | NA | LR-LC |
| ST_B_CELL_ANTIGEN_RECEPTOR | 0 | 0 | NA | NA | LR-LC |
| HSA00562_INOSITOL_PHOSPHATE_METABOLISM | 1 | -1 | NA | NA | LR-LC |
| HSA02010_ABC_TRANSPORTERS_GENERAL | -1 | 1 | NA | NA | LR-LC |
| TPOPATHWAY | 0 | 0 | NA | NA | LR-LC |
| MRNA_PROCESSING_REACTOME | -2 | 2 | NA | NA | MR-LC |
| HSA04810_REGULATION_OF_ACTIN_CYTOSKELETON | 0 | 0 | NA | NA | LR-LC |
| SIG_CD40PATHWAYMAP | 2 | -2 | NA | NA | LR-LC |
| GSK3PATHWAY | 0 | 0 | NA | NA | LR-LC |
| HSA00150_ANDROGEN_AND_ESTROGEN_METABOLISM | 1 | -1 | NA | NA | LR-LC |
| IGF1MTORPATHWAY | 0 | 0 | NA | NA | LR-LC |
| PYK2PATHWAY | 0 | 0 | NA | NA | LR-LC |
| KERATINOCYTEPATHWAY | 0 | 0 | NA | NA | LR-LC |
| HSA04010_MAPK_SIGNALING_PATHWAY | 0 | 0 | NA | NA | LR-LC |
| TNFR1PATHWAY | -2 | 2 | NA | NA | LR-LC |
| HSA04664_FC_EPSILON_RI_SIGNALING_PATHWAY | 0 | 0 | NA | NA | LR-LC |
| LCALPAINPATHWAY | 1 | -1 | NA | NA | LR-LC |
| FCER1PATHWAY | 1 | -1 | NA | NA | LR-LC |
| ST_WNT_BETA_CATENIN_PATHWAY | 0 | 0 | NA | NA | LR-LC |
| SIG_PIP3_SIGNALING_IN_CARDIAC_MYOCTES | 0 | 0 | NA | NA | LR-LC |
| UREA_CYCLE_AND_METABOLISM_OF_AMINO_GROUPS | 0 | 0 | NA | NA | LR-LC |
| HSA00020_CITRATE_CYCLE | 1 | -1 | NA | NA | MR-LC |
| ATP_SYNTHESIS | -2 | 2 | NA | NA | LR-LC |
| FMLPPATHWAY | 0 | 0 | NA | NA | LR-LC |
| TYPE_III_SECRETION_SYSTEM | -2 | 2 | NA | NA | LR-LC |
| HSA00670_ONE_CARBON_POOL_BY_FOLATE | -2 | 2 | NA | NA | LR-LC |
| HSA00590_ARACHIDONIC_ACID_METABOLISM | 0 | 0 | NA | NA | LR-LC |
| HSA04660_T_CELL_RECEPTOR_SIGNALING_PATHWAY | 0 | 0 | NA | NA | LR-LC |
| P53HYPOXIAPATHWAY | 1 | -1 | NA | NA | LR-LC |
| TCRPATHWAY | 0 | 0 | NA | NA | LR-LC |
| ARAPPATHWAY | 0 | 0 | NA | NA | LR-LC |
| RARRXRPATHWAY | 0 | 0 | NA | NA | LR-LC |
| SIG_PIP3_SIGNALING_IN_B_LYMPHOCYTES | 0 | 0 | NA | NA | LR-LC |
| ST_FAS_SIGNALING_PATHWAY | -2 | 2 | NA | NA | MR-LC |
| HSA03022_BASAL_TRANSCRIPTION_FACTORS | 0 | 0 | NA | NA | LR-LC |
| HSA04115_P53_SIGNALING_PATHWAY | 0 | 0 | NA | NA | LR-LC |
| HSA04310_WNT_SIGNALING_PATHWAY | 1 | -1 | NA | NA | LR-LC |
| SPPAPATHWAY | 2 | -2 | NA | NA | LR-LC |
| CERAMIDEPATHWAY | -2 | 2 | NA | NA | LR-LC |
| CREBPATHWAY | 0 | 0 | NA | NA | LR-LC |
| FRUCTOSE_AND_MANNOSE_METABOLISM | 1 | -1 | NA | NA | LR-LC |
| PROSTAGLANDIN_AND_LEUKOTRIENE_METABOLISM | 0 | 0 | NA | NA | LR-LC |
| HSA00340_HISTIDINE_METABOLISM | -1 | 1 | NA | NA | LR-LC |
| MPRPATHWAY | 1 | -1 | NA | NA | LR-LC |
| STRESSPATHWAY | -1 | 1 | NA | NA | LR-LC |
| ETSPATHWAY | 0 | 0 | NA | NA | LR-LC |
| NGFPATHWAY | 0 | 0 | NA | NA | LR-LC |
| RACCYCDPATHWAY | 0 | 0 | NA | NA | LR-LC |
| HSA04510_FOCAL_ADHESION | 2 | -2 | NA | NA | MR-LC |
| HSA04540_GAP_JUNCTION | 0 | 0 | NA | NA | LR-LC |
| SIG_REGULATION_OF_THE_ACTIN_CYTOSKELETON_BY_RHO_GTPASES | -2 | 2 | NA | NA | LR-LC |
| G1PATHWAY | 0 | 0 | NA | NA | LR-LC |
| IL2PATHWAY | 0 | 0 | NA | NA | LR-LC |
| ERK5PATHWAY | 0 | 0 | NA | NA | LR-LC |
| P38MAPKPATHWAY | 0 | 0 | NA | NA | LR-LC |
| PEPTIDE_GPCRS | 0 | 0 | NA | NA | LR-LC |
| ST_GAQ_PATHWAY | 0 | 0 | NA | NA | LR-LC |
| CALCIUM_REGULATION_IN_CARDIAC_CELLS | 0 | 0 | NA | NA | MR-LC |
| CXCR4PATHWAY | 1 | -1 | NA | NA | LR-LC |
| HSA04912_GNRH_SIGNALING_PATHWAY | 1 | -1 | NA | NA | LR-LC |
| VEGFPATHWAY | -2 | 2 | NA | NA | LR-LC |
| ARGININE_AND_PROLINE_METABOLISM | 0 | 0 | NA | NA | LR-LC |
| IL2RBPATHWAY | 0 | 0 | NA | NA | LR-LC |
| HSA04320_DORSO_VENTRAL_AXIS_FORMATION | 0 | 0 | NA | NA | LR-LC |
| PHOSPHATIDYLINOSITOL_SIGNALING_SYSTEM | 0 | 0 | NA | NA | LR-LC |
| CARM_ERPATHWAY | 1 | -1 | NA | NA | LR-LC |
| EIF4PATHWAY | 0 | 0 | NA | NA | LR-LC |
| ST_G_ALPHA_I_PATHWAY | 0 | 0 | NA | NA | LR-LC |
| HSA04630_JAK_STAT_SIGNALING_PATHWAY | -2 | 2 | NA | NA | MR-LC |
| HSA04060_CYTOKINE_CYTOKINE_RECEPTOR_INTERACTION | 2 | -2 | NA | NA | MR-LC |
| HSA04330_NOTCH_SIGNALING_PATHWAY | 2 | -2 | NA | NA | LR-LC |
| HSA00530_AMINOSUGARS_METABOLISM | 1 | -1 | NA | NA | LR-LC |
| HSA04350_TGF_BETA_SIGNALING_PATHWAY | 0 | 0 | NA | NA | LR-LC |
| GPCRPATHWAY | 2 | -2 | NA | NA | LR-LC |
| HSA04150_MTOR_SIGNALING_PATHWAY | -2 | 2 | NA | NA | LR-LC |
| HSA04620_TOLL_LIKE_RECEPTOR_SIGNALING_PATHWAY | 0 | 0 | NA | NA | LR-LC |
| ST_JNK_MAPK_PATHWAY | 0 | 0 | NA | NA | LR-LC |
| HSA04360_AXON_GUIDANCE | 0 | 0 | NA | NA | LR-LC |
| ST_PHOSPHOINOSITIDE_3_KINASE_PATHWAY | 0 | 0 | NA | NA | LR-LC |
